# Assessing Outlier Probabilities in Transcriptomics Data When Evaluating a Classifier

**DOI:** 10.3390/genes14020387

**Published:** 2023-02-01

**Authors:** Magdalena Kircher, Josefin Säurich, Michael Selle, Klaus Jung

**Affiliations:** Institute for Animal Breeding and Genetics, University of Veterinary Medicine Hannover, D-30559 Hannover, Germany

**Keywords:** bootstrap, bagplot, outlier detection, outlier probability, robust learning, transcriptomics data

## Abstract

Outliers in the training or test set used to fit and evaluate a classifier on transcriptomics data can considerably change the estimated performance of the model. Hence, an either too weak or a too optimistic accuracy is then reported and the estimated model performance cannot be reproduced on independent data. It is then also doubtful whether a classifier qualifies for clinical usage. We estimate classifier performances in simulated gene expression data with artificial outliers and in two real-world datasets. As a new approach, we use two outlier detection methods within a bootstrap procedure to estimate the outlier probability for each sample and evaluate classifiers before and after outlier removal by means of cross-validation. We found that the removal of outliers changed the classification performance notably. For the most part, removing outliers improved the classification results. Taking into account the fact that there are various, sometimes unclear reasons for a sample to be an outlier, we strongly advocate to always report the performance of a transcriptomics classifier with and without outliers in training and test data. This provides a more diverse picture of a classifier’s performance and prevents reporting models that later turn out to be not applicable for clinical diagnoses.

## 1. Introduction

High-throughput transcriptomics data, taken by DNA microarrays [1] or RNA-Seq [2], are regularly used for training classifier models, e.g., for the purposes of medical diagnosis, prediction, and prognosis [3,4,5]. In the last twenty-five years, transcriptomic classifier models have been presented for a large spectrum of medical classification problems, including diagnosis of cancer [6], infectious diseases [7], or neurological disorders [8]. When only one dataset is available, model performance measures such as accuracy, sensitivity (‘recall’), and specificity are usually first assessed within these training data by leave-one-out-, *k*-fold-, or bootstrap cross-validation [9,10]. To additionally confirm a classifier’s performance, independent test data are required, because validation solely on the training data often yields a too optimistic accuracy estimate [11]. It was also observed that many published classifier models based on high-throughput gene expression data were not ready for clinical use [12].

One reason for the differences between accuracies obtained from within training data validation and from validation in external test data are divergences in the distribution of sample characteristics. Simple examples would be the male/female ratio or the age distribution is different in the training and test data. In addition to differences in the distribution of sample characteristics, batch and study effects in the expression data themselves can result in different outcomes of classifier evaluations from within the training data or from the test data [13,14]. The problem of study effects in classifier evaluation can, for example, be solved by cross-study instead of leave-one-out cross-validation [15].

The difference in the distribution of sample characteristics in the training and test data can especially be affected by outliers in one or both datasets. Outliers, basically samples with extreme data, can either be caused technically (e.g., measuring errors, mislabeling) or represent real biological extremes. In the case of a classification problem with a pre-defined number of classes, an outlier can also represent an unspecified additional class or an unspecified subpopulation [16]. In infection research, outliers in transcriptomics data, for example, can also be caused by undetected co-infections [17]. Outliers in the training or test data, independent of their origin, can cause a decrease or increase in a classifier’s accuracy. Depending on the location of an outlier, the separability of classes may become easier or more difficult.

Outliers also play a role in the detection of differentially expressed genes that are to be selected as predictor variables for the classifier models. Many microarray and RNA-Seq studies have focused on finding such subsets of genes, often referred to as ‘molecular signatures’ [18,19]. However, when comparing the results of studies on differential expression analysis in the same disease, the overlap of differentially expressed genes from each study is rather small [20]. Hence, also depending on whether extreme observations are removed or retained, different sets of top *N* significant genes will most likely be found in the differential gene expression analysis.

Moreover, it is difficult to determine how much a data point must deviate from the remaining data points, based on a certain distance measure, to be considered an outlier, especially in high-dimensional data such as gene expression data [21]. A number of approaches have been proposed to detect outlying samples in transcriptomics data, usually in the step of data exploration after dimension reduction by principal component analysis (PCA). Proposed methods involve robust PCA for single groups [22], PCA coupled with bagplots for multi-group outlier detection [23], and a PCA-Grid approach [24]. When outliers have been detected, however, it is often hard to decide whether outliers should be removed or kept for the downstream analysis. A typical step is usually to discuss an outlying sample with the partner of the microarray or RNA-Seq facility who generated the data whether these samples could be technical outliers, and with the clinical partner who provided the sample material from a patient whether this patient has been properly allocated to the respective study group. This step includes, however, the risk of biased views from the contributing researchers. Therefore, we are arguing here that it is very important to determine classifier performance using data before and after outlier removal to obtain a holistic impression of the range of the accuracy and other performance measures. Although standard cross-validation procedures also take subsets with and without outliers into account, performance measures are reported as averages over all cross-validation runs. In contrast, we present here an approach that provides a separate view on the classifier performance with all samples compared to the classifier performance after outlier removal. Thus, our approach allows for the study of more extreme scenarios yielding a wider range of estimated classifier performance.

To facilitate the handling of outliers, we present a new algorithm for determining outlier probabilities for each sample using a bootstrap approach. Bootstrap approaches have a long tradition in bioinformatics to study the distribution of analysis results [25,26,27]. Researchers can then decide to include or exclude outliers for classifier evaluation based on these probabilities. Furthermore, we demonstrate on simulated and two real-world datasets how outliers can change the performance estimates of a classifier model.

## 2. Materials and Methods

**Figure 1:**
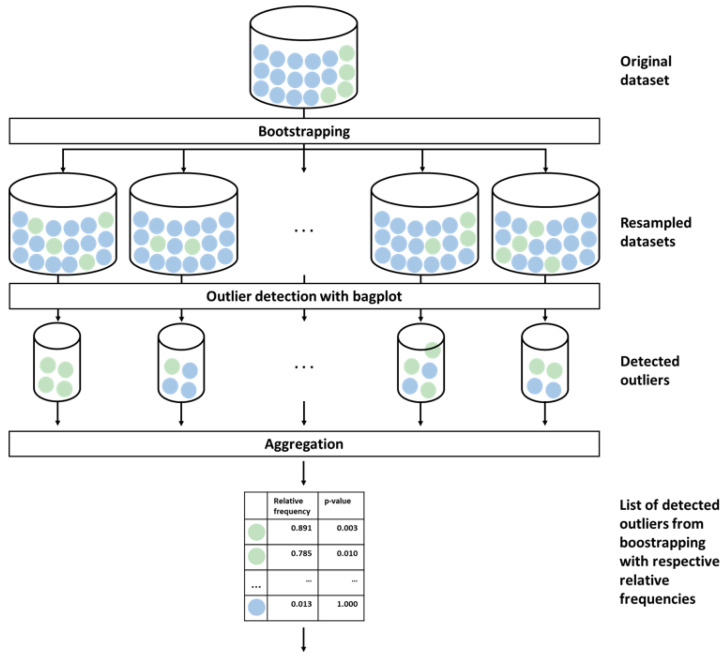
First part of the analysis pipeline: resampling and outlier detection. In the first step, the original dataset is resampled *B* times using random sampling with replacement. For each resampled dataset, outlier detection is conducted for the two study groups separately using bagplot or PCA-Grid algorithm. The samples marked as outliers for each bootstrap run were finally aggregated into relative frequencies, i.e., outlier probabilities. In addition, the *p*-value of the binomial test is reported.

**Figure 2:**
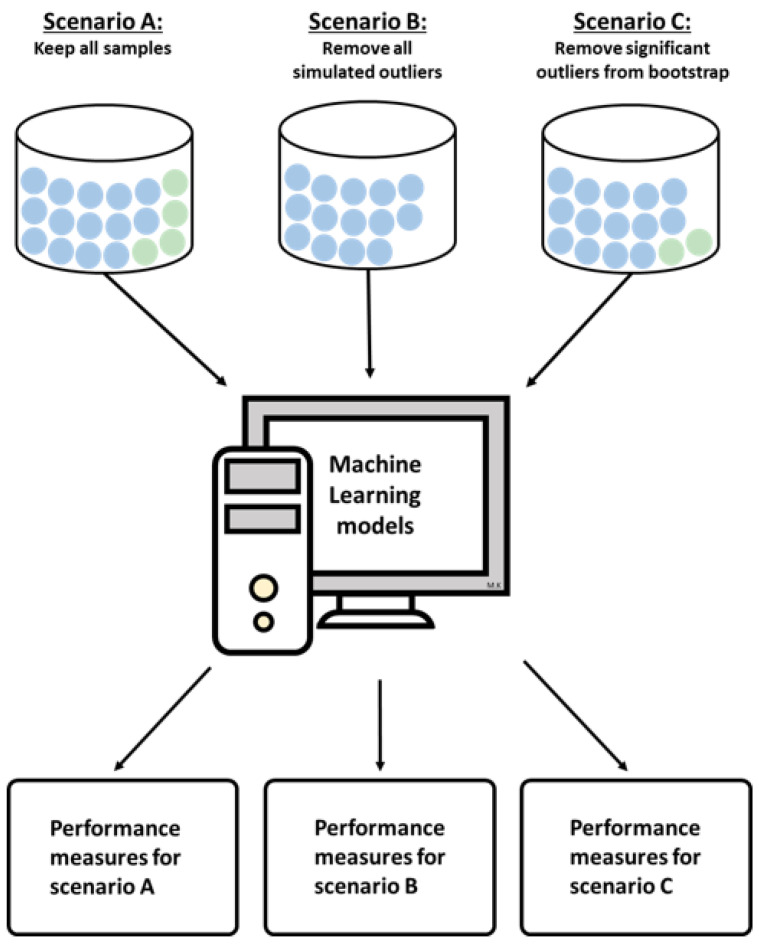
Second part of the analysis pipeline: evaluation of model performances for different outlier handling scenarios. After assessing outlier probabilities, three different scenarios were tested for the simulation data: (**A**) keep all samples, (**B**) remove all simulated outliers, (**C**) remove samples that were identified as significant outliers after bootstrapping. For all scenarios, three different ML models were trained and tested. Performance measures for different scenarios, ML models, and numbers of included genes were reported.

In this section, we describe the standard procedures of training and evaluating the machine learning (ML) models, our new procedure for assessing outlier probabilities, as well as the simulated and real-world data examples. We performed all analyses using the R programming environment (www.r-project.org (accessed 5 May 2022), version 4.1.2). Figure 1 and Figure 2 show the workflow of resampling, outlier detection, and cross-validation of the models for the different scenarios regarding the treatment of the detected outliers. Figure 1 shows the first part of the analysis pipeline, including bootstrapping and outlier detection. Using bootstrap, newly resampled datasets are drawn from the original data repeatedly. For each resampled dataset, potential outliers in both study groups are identified using bagplots. By aggregating the frequencies with which the samples were represented in bootstrap datasets and the frequencies with which the samples were detected as outliers, relative outlier frequencies, i.e., outlier probabilities, could be calculated. A more detailed description of the method for calculating the outlier probability is given in Section 2.2. Figure 2 shows the second part of the analysis pipeline, which is the comparison of the different outlier handling strategies: (A) retain all samples including outliers, (B) remove all simulated outliers, and (C) remove samples that were identified as significant outliers using our proposed method. The outcomes for different classifiers were assessed using cross-validation. In addition, the models were tested for various numbers of top differentially expressed genes. A more precise explanation of the methods used to compare the different outlier handling strategies will be given in the following subsection.

### 2.1. Feature Selection, Classification Models, and Bootstrap Validation

When training and evaluating classification models with high-dimensional data, feature selection is a necessary step to reduce the risk of overfitting. Feature selection means to select a subset of predictor genes as a molecular signature. Therefore, differential gene expression analysis was conducted using the ‘limma’ package in R to determine the most significantly differentially expressed genes between two groups of samples to be included in the classifier models. An incremental feature selection was carried out by ranking the genes according to their significance, in descending order. The models were then evaluated by successively increasing the number of predictor genes from 10 to 200 by steps of 10.

We evaluated each data scenario using three different classification methods, namely support vector machine (SVM), random forest (RF), and linear discriminant analysis (LDA). These three ML models have widely been used in the context of high-dimensional expression data ([28,29,30]). The SVM models were fitted using the default settings of the R-package ‘e1071’, i.e., with linear kernels and a cost of constraints violation of 10. The RF models, fitted using the R-package ‘randomForest’ [31], consisted of 500 trees, and the number of variables tried at each split was set to the square root of the number of included variables. LDA models were also performed with default settings in the R-package ‘MASS’ [32].

To determine how well the trained models generalize on new data, bootstrap validations were conducted, i.e., random subsets consisting of 2/3 of all samples were used for training a model, and the respective hold-out subset was used for testing. The training subset was constructed by sampling without replacement from the original data. Accordingly, the test dataset consisted of the remaining third of the observations. Model performance was assessed by classification accuracy, sensitivity (recall), specificity, and positive (precision) and negative predictive values. In addition, the Brier score was assessed. Whereas the accuracy reflects the proportion of correct classifications, the Brier score reflects the mean difference between true classes and predicted class probabilities [33]. This bootstrap procedure was repeated 100 times to get robust estimates of the models’ performances. Finally, the mean of the aforementioned performance measures was determined over the 100 runs.

### 2.2. Assessment of Outlier Probabilities

Outlier probabilities were quantified using a separate bootstrap procedure, specifically by resampling one dataset 100 times with replacement and detecting the outliers in each resampled dataset. For the outlier detection itself, we used first the bagplot algorithm [34], which is a bivariate extension of the boxplot and is suitable for molecular high-throughput data after dimension reduction [23]. The bagplot was applied separately to the point cloud of each study group in the two-dimensional space of principal components. The bagplot was computed using the identically named function of the R-package ‘aplpack’ [35] and with a factor of two, which influences the extension of the ‘bag’ and hence affects the threshold at which a data point is defined as an outlier. As a second approach, we used the so-called PCA-Grid approach [24], where principal components are determined in a robust manner, and outliers are detected by analyzing distances to the center of the point cloud. This approach was implemented using the R-packages ‘pcaPP’ [36] and ‘rrcov’ [37], respectively. Next, over all 100 bootstrap runs, we counted the frequency of each sample to be selected as an outlier and used the relative frequencies as outlier probabilities. Based on the absolute frequencies, a binomial test was run per sample to test whether the outlier probability of this sample was significantly larger than 50%. A *p*-value of 0.05 was used as the significance level.

The formal procedure for determining outlier probabilities and binomial test results can be summarized as shown in the following pseudo-code (Algorithm 1), here for the bagplot approach. The algorithm for the PCA-Grid method works analogously. A new R-function ‘bagplotol’, presented in the Supplementary R-code implements this bootstrap algorithm, and a new R-function ‘pcagridol’ implements the bootstrap approach for the PCA-Grid method. Arguments of both R-functions are the number of bootstrap runs as well as two matrices of normalized expression levels.
**Algorithm 1** Pseudo-code of bootstrap–bagplot algorithm for outlier detection for two study groups.1:Initialize lists to report the samples that are randomly drawn from the original dataset *D* in the bootstrap runs and the outliers that are detected in each case:    Samples=[]    Outliers=[]2:**for**i=1,2,…,I**do**3:    Create a bootstrap dataset $B_{i}$ by resampling the original dataset *D* with replacement.4:    Report the sample IDs present in $B_{i}$, i.e., $Samples_{B_{i}}$ with        $Samples=append(Samples,Samples_{B_{i}})$.5:    Conduct a principal component analysis for $B_{i}$ including samples from both study groups.6:    Split $B_{i}$ into the two study group subsets $B_{i1}$ and $B_{i2}$.7:    **for** k=1,2 **do**8:        Detect outliers of *k*-th study group subset $B_{ik}$ using bagplot:            $Outliers_{B_{ik}}=bagplot\left(B_{ik},factor=2\right)$9:        Report the samples being detected as outliers with            $Outliers=append(Outliers,Outliers_{B_{ik}})$10:    **end for**11:    Aggregate Samples list to frequency table.12:    Aggregate Outliers list to frequency table.13:    Compute relative outlier frequencies and conduct binomial test with α=0.05.14:**end for**

### 2.3. Simulated and Real-World Example Data

For evaluation, we first constructed simulated data to study the effect of outliers under a controlled setting and used additional real-world data for illustration. In all scenarios, a two-study-groups design was used. In general, the simulated and the real-world data represented scenarios with different numbers of transcripts, different sample sizes, and different proportions of outliers.

Table 1 shows a general overview of the datasets used for the evaluation. A more detailed description on the simulation data and the two real-world datasets is given in the following subsections.

#### 2.3.1. Simulated Data

We studied simulation scenarios for the cases of two and four study groups, respectively, and generated data for 1000 genes in both scenarios. For both cases of two and four study groups, we generated data representing gene expression values for 50 samples per study group. In addition, five outlier samples per group were generated. Hence, 110 and 220 samples were simulated in total for the two-study-group and the four-study-group design, respectively. In the following, we describe the procedure for generating the data for the case of two study groups.

To obtain different distributions for the two study groups, differential expression was set by providing a non-zero log2 fold change between the two phenotypes for a specified number of transcripts. Following [38], a baseline mean $\mu_{t}$ was determined for each transcript *t* (t=1,…,d), and the group specific means $\mu_{t1}$ and $\mu_{t2}$ were both set to $\mu_{t}$. Then, if the log2 fold change $\lambda_{t}$ was provided for a certain transcript, the mean for the second group was changed as follows: $\mu_{t2}$ = $\mu_{t1}$*$2^{\lambda_{t}}$. The expression data were then drawn for each transcript from the normal distribution with mean $mu_{tk}$ (k=1,2). In the same way, we changed mean expression levels within both groups to obtain mean vectors for generating outlier data.

It should be noted that for most mean transcript values no fold change was determined between the groups to keep separability moderate. Otherwise, overly large differences would have led to an oversimplification of the classification of the groups for the ML models. Accordingly, we constructed the outlier samples such that a sample was not detected in all bootstrap runs as an outlier. We present example R-code as Supplementary Material to depict the procedures of data generation in detail.

#### 2.3.2. SARS-CoV-2 versus Other Respiratory Viruses

The first real-world dataset we used for illustrating our findings from the simulation study was taken from a study on the effect of SARS-CoV-2 infections on the transcriptome [39]. The authors of this study also took the transcriptome of human patients with other respiratory diseases and fitted a classifier to distinguish the two groups. We used a subset of these data to study the effect of outliers on the estimated model performance. The whole dataset is available from the Gene Expression Omnibus (GEO) database under the accession number GSE163151. Our selected subset consists of 138 samples in the SARS-CoV-2 group and 120 samples in the group of other viral respiratory diseases. Original data were available as read counts for 26,485 genes from high-throughput sequencing. We filtered 1633 genes with a low expression level, i.e., genes with maximum counts per million less than 1. Read counts were transformed using the ‘voom’ function of the R-‘limma’-package to obtain continuous and normalized data. The two-study-groups scenario we used here was as follows: the class ‘COVID’ contained expression data from nasopharyngeal swabs from all 138 patients being infected with SARS-CoV-2 and the class ‘other virus’ consisted of expression data from nasopharyngeal swabs from 120 patients with respiratory disease caused by other virus infections.

#### 2.3.3. West-Nile Infected versus Control Samples

The second real-world scenario is from a study on responses to infection with West Nile virus (WNV) [40]. In the original study, the authors investigated, among other aspects, if the history of WNV infection being either asymptomatic or severe affects the response to re-infection with WNV. For this purpose, peripheral blood mononuclear cells (PBMCs) and macrophages in particular were analyzed with respect to their expression profile. The cohort for the microarray analysis consisted of 21 human donors with previous asymptomatic courses of WNV infection and 18 human donors with previous severe courses of WNV infection. PBMCs from all donors were then each incubated in medium alone (‘mock’) as well as infected with WNV ex vivo to see if the gene expression profiles of the two study groups varied considerably for different treatments of the cells. In this analysis, only one gene was reported for which differences were found between the groups ‘asymptomatic course with WNV infection in the past’ and ‘severe course with WNV infection in the past’ when PBMCs were infected with WNV ex vivo with regard to gene expression values. Overall, the expression levels for 47,323 transcripts were available. The original dataset also contains microarray data from PBMCs stimulated with poly(I:C) and microarray data from macrophages and is available from the GEO database under the accession number GSE46681.

In contrast to the original study, we pooled the samples of patients who had a previous asymptomatic course or a severe course of disease after infection with WNV. We then contrasted the PBMC expression profiles from the whole group for the two conditions ‘mock treatment’, i.e., negative control, and ‘WNV infection ex vivo’ to study the effect of outliers on the estimated model performance. Hence, the two-study-groups scenario was as follows: the class ‘mock’ contained expression data from PBMCs from all 39 donors that were incubated in medium alone, and the class ‘WNV’ consisted of expression data from PBMCs from all 39 donors that were infected with WNV ex vivo.

## 3. Results

In this section, we first describe the results of the simulation study and next the effects of outlier identification and removal on the classification performance in the two real-world datasets. In the two subsections on the results with the two real-world datasets, strategy B (removal of all simulated outliers) was, of course, not studied.

### 3.1. Classifier Evaluation in Simulated Data

**Figure 3:**
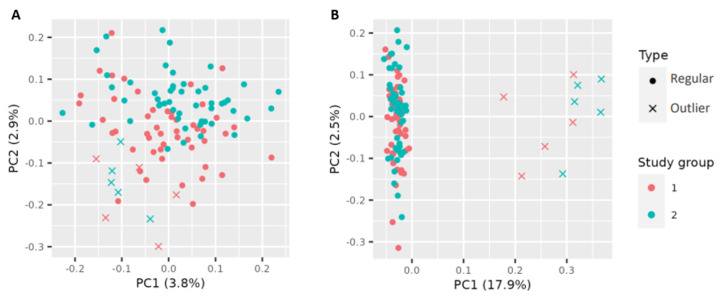
Examples of sample distributions in the space of the first two principal components from two selected simulation runs. In each simulation run, 50 samples per study group were generated plus 5 outlier samples per group. In some simulation runs, outliers were close to the regular samples of the study groups (**A**), and in other runs, outliers were more distant from the true groups (**B**).

First, simulation data with typical characteristics of gene expression data were generated to test the outlier detection method, including calculation of outlier probability and its influence on classification performances in a controlled setting. To mimic data for the two-group study design, differential expression was set by providing a non-zero log fold change between the two phenotypes for some transcripts, as described in Section 2.3.1. Between the two study groups, for 100 randomly selected genes, the log fold change was set as non-zero, and for the outlier groups, 50 genes were randomly selected to have a non-zero fold change. This data construction setting was repeatedly used to generate differently distributed simulation datasets. More precisely, in total 100 simulation runs with varying data distributions were carried out. Figure 3 shows the data after dimension reduction by PCA for two simulation runs with this setting. It happened that sometimes, the outliers were less extreme (A) or more clear (B).

For each simulated dataset, i.e., for each of the 100 simulation runs, our proposed bootstrap approach for bagplot and PCA-Grid was used to assess the respective outlier probabilities, as described in Section 2.2.

**Figure 4:**
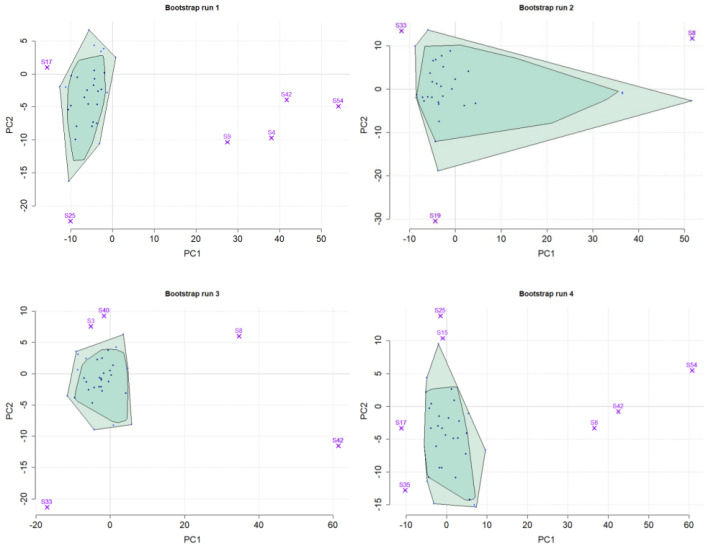
Four (out of 100) bootstrapped datasets from one simulation run in the PCA space with bagplots for outlier detection. The figure shows bootstrap samples always from the same study group. It can be seen in the selected example runs that not all outliers are detected in each bootstrap run. This may be because a simulated outlier was not selected for the respective bootstrap dataset or because the presence or absence of other samples in the bootstrap dataset prevents the outlier from being detected as such. In the same way, regular samples can be detected as outliers. The simulated outliers for study group 2 in this simulation run were ‘S4’, ‘S8’, ‘S9’, ‘S42’, and ‘S54’.

Figure 4 shows bagplots with the corresponding outliers for the second study group in one simulation run for four different bootstrap runs. As the plots show, the detection or non-detection is affected by the composition of the samples in the respective bootstrap dataset. By reporting the times each sample was detected as an outlier, we received a robust estimate for its respective outlier probability.

In each of 100 simulation runs, we determined the performance measures for the three different classifier models, when either keeping all samples (strategy A), when removing the simulated outliers (‘true outliers’) (strategy B), or when removing only the samples detected as outliers by our proposed bootstrap method (strategy C). For the cases where no significant outlier was detected, the same data were used for strategy A and strategy C, and consequently the same performance values were reported. This was, for example, the case in 10 of 100 simulation runs when using bagplot for outlier detection.

Performance values were always taken in dependence of the number of differentially expressed genes selected as predictors in the ML algorithms. The different ML models used for classification were also cross-validated by randomly drawing 2/3 of the original data as training samples without replacement for 100 times. In each cross-validation run, the remaining 1/3 of samples were used as test data to evaluate the performance of the previously trained classifiers.

**Figure 5:**
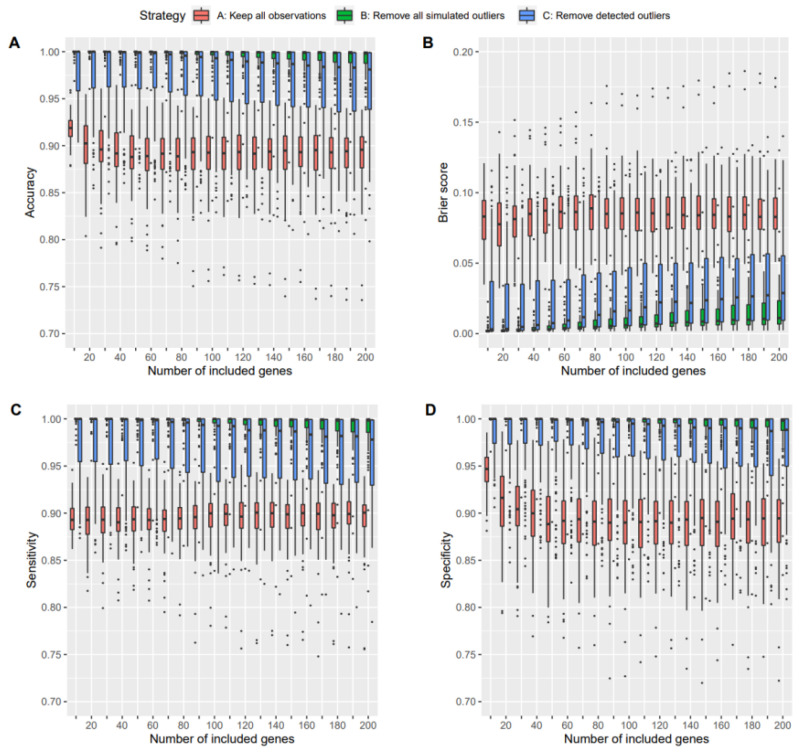
Performances for different outlier handling strategies. The plots show distributions of mean accuracies (**A**), mean Brier scores (**B**), mean sensitivities (**C**), and mean specificities (**D**) over 100 simulation runs, respectively, when using SVMs for classification and for different numbers of top differentially expressed genes to be included as predictors in the models. Strategy A was generally outperformed by B and C.

For all ML methods and for all performance measures, we observed that the performance was increased when outliers were removed. Furthermore, removing all true outliers yielded even higher performance than removing only the detected outliers. The plots in Figure 5 show the distributions of mean values for accuracies, Brier scores, sensitivities, and specificities obtained for the different outlier handling strategies over the 100 simulation runs when using SVMs for classification and bagplot as outlier detection algorithm. The results for the other performance measures, such as negative and positive predictive values, and for the other classifier models, including LDA and RF, are provided as Supplementary Figures. We observed similar results for LDA and RF models, except that the LDA models provided overall less performance than SVM and RF models. The overall outcome of the comparison between strategies A, B, and C was more or less the same when using either the bagplot or PCA-Grid technique. The comparisons between these two techniques are shown as part of the Supplementary Figures.

During the analysis of our simulation data, we considered that the presence of outliers, depending on where they are located relative to the regular observations of the two groups, can facilitate the classification. In other words, we assumed that the location of outliers can either increase or decrease the separability of two groups. Therefore, we studied the classifier performances within each simulation run in more detail. In particular, we inspected the distribution of the samples of each simulation run and the corresponding classification results.

**Figure 6:**
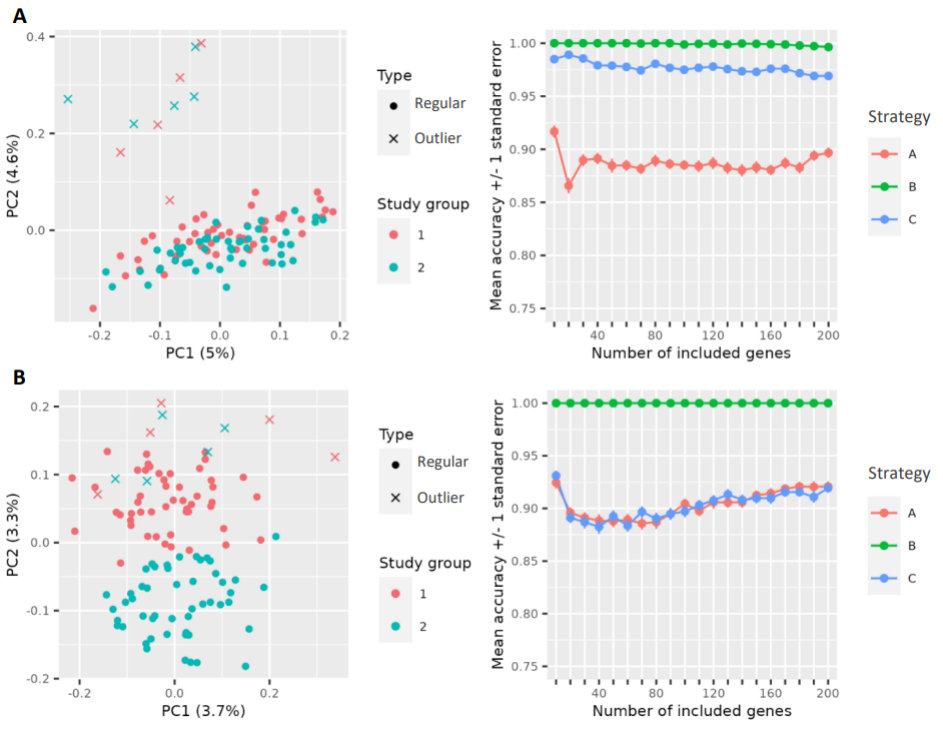
Two exemplary simulated datasets with associated classification outcomes for different scenarios regarding the handling of outliers represented by mean accuracies +/− standard errors. For the simulation data displayed in plot (**A**), seven outliers in total were identified with the bootstrap–bagplot approach and removed in strategy C (blue line), respectively. For the simulation displayed in the plot below (**B**), only one sample was detected and removed for strategy C.

Figure 6 shows the data distributions in the PCA space from two simulation runs with associated classifier performances, i.e., accuracy measures using SVM as classifier method. In plot A, outliers of both groups are located close to each other aside to the regular observations of the groups. From the perspective of the first PC, the outliers are lying between the two groups, hence they hamper the separability of the two groups. Their removal therefore increases the accuracy. In plot B, again the outliers of both groups are close to each other but also located within the regular observations of study group 1. Thus, the outliers of group 1 would eventually facilitate the classification, whereas the outliers of group 2 would hamper the classification. However, the removal of all simulated outliers clearly increased the accuracy. For the simulation data shown in plot A, three samples from the first group and four samples from the second study group were identified as significant outliers using the bootstrap–bagplot algorithm, whereby one identified outlier in the first group was not one of the simulated outliers. In the simulation scenario of plot B, only one sample from study group 1 was identified as an outlier by the bootstrap algorithm, and therefore, strategy A and C yielded similar accuracies.

For the four-study-group scenario, the outlier probability estimation was conducted similarly to the two-study-group design. The proposed bootstrap method using both PCA-Grid and bagplot algorithm, respectively, successfully detected potential outliers regarding their occurrences during bootstrapping. When evaluating the classifiers, it could be observed that the accuracy increased until the number of genes was 30 and decreased with further numbers of genes included as predictor variables in the models. With an increasing number of genes above 30, slightly lower accuracies were observed when all simulated outliers were excluded from the dataset, compared to strategy A, where all samples were included in the models. Between strategy A and strategy C, i.e., only samples detected by our proposed method were removed, no substantial difference could be observed regarding accuracy and Brier score.

### 3.2. Classifier Evaluation in SARS-CoV-2 Study

For the SARS-CoV-2 dataset, the outlier probabilities and the classification performances for the different outlier handling strategies were assessed in the same way as for the simulation data. Similarly, the performances at different numbers of top significantly differentially expressed genes as predictors were reported.

Supporting the findings of the simulation study, it could also be shown for the SARS-CoV-2 data that the detection of outliers was significantly affected by the composition of the samples present in the respective bootstrap dataset. Of all 258 samples, 77 were detected as outliers in at least one bootstrap run, but in total only 12 had an outlier probability significantly larger than 0.5. Using the bootstrap approach for PCA-Grid, 26 samples were identified as outliers, whereby 9 of the samples were also found within the aforementioned bagplot method.

Table 2 shows the 20 samples most frequently detected as outliers using the bootstrap–bagplot algorithm. For each of these samples, we present the number of occurrences in the 100 bootstrap runs, the number of bootstrap runs where a sample was detected as an outlier, the estimated outlier probability with a 95%-confidence interval, as well as *p*-values from the binomial test. For example, the sample ‘GSM4972835’ occurred in 68/100 bootstrap runs and was detected as an outlier in all 68 runs. The estimated outlier probability therefore is 1.00. The 12 outlying samples with an outlier probability significantly larger than 0.5 include six samples from the group ‘other Virus’ and six samples from the group ‘SARS-CoV-2’. Analogously, the most frequently found outliers of the PCA-Grid method are reported as a Supplementary Table S1.

**Figure 7:**
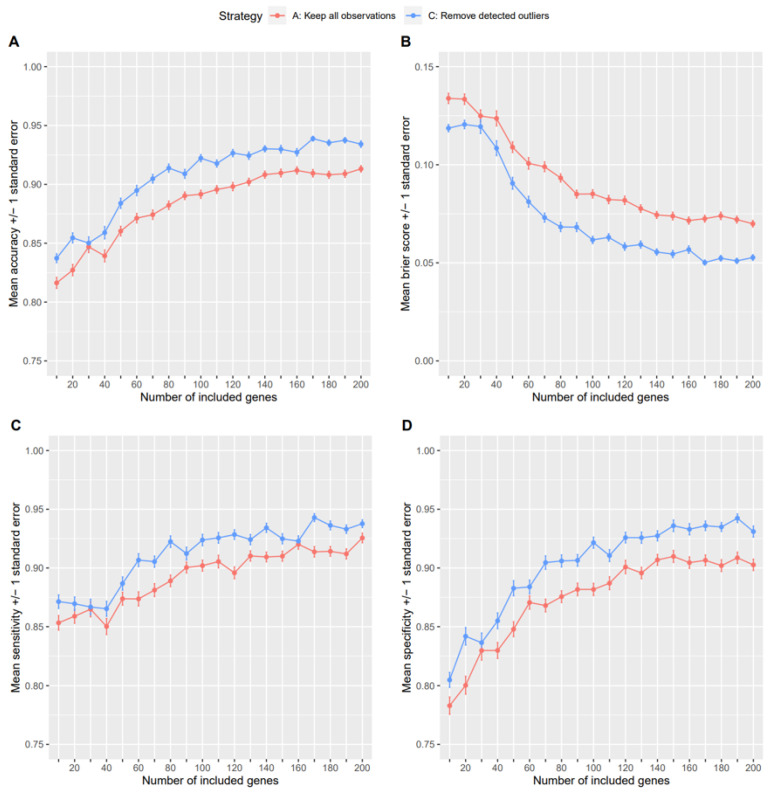
Performance measures for different outlier handling strategies for the SARS-CoV-2 dataset. The plots show mean values with bars representing standard errors for accuracy (**A**), Brier score (**B**), sensitivity (**C**), and specificity (**D**), respectively, when using a SVM for classification and for different numbers of most differentially expressed genes to be included in each of the models.

The removal of samples with outlier probabilities significantly higher than 0.5 led to different classification results than when all samples were kept in the dataset. Figure 7 presents mean (+/− standard error) accuracy, Brier score, sensitivity, and specificity from 100 cross-validation runs in dependence of the number of genes included as predictor variables. The figure shows the results for classification using SVMs as the ML approach. For all four measures, strategy C, in which the detected outliers were removed, consistently showed better performances than strategy A, in which all samples were kept. Results for positive and negative predictive values and when using LDAs and RFs are provided as Supplementary Material. There again, strategy C yields a higher classification performance in nearly all scenarios.

### 3.3. Classifier Evaluation in West-Nile Virus Study

For the WNV dataset, the evaluation was carried out the same way as for the SARS-CoV-2 data. Analogous to the SARS-CoV-2 study, the bagplot–bootstrap procedure was used to detect any outliers and to assess outlier probabilities. Subsequently, the classification performances for the different classification models using SVMs, RFs, and LDAs were calculated for different numbers of genes included in the models.

Table 3 shows the samples being detected in some bootstrap runs using bagplots including their estimated outlier probabilities and *p*-values for the binomial test. Overall, only one sample was found to be a significant outlier. The respective sample belonged to the group of PBMCs infected with WNV. All other samples shown in the table had an outlier probability of less than 0.5. Using PCA-Grid technique within the bootstrap runs led to two samples identified as significant outliers, i.e., one sample from each study group. The sample identified as an outlier when using bagplot was not counted as an outlier. A table of most frequently found outliers from the PCA-Grid method is included in the Supplementary Materials.

**Figure 8:**
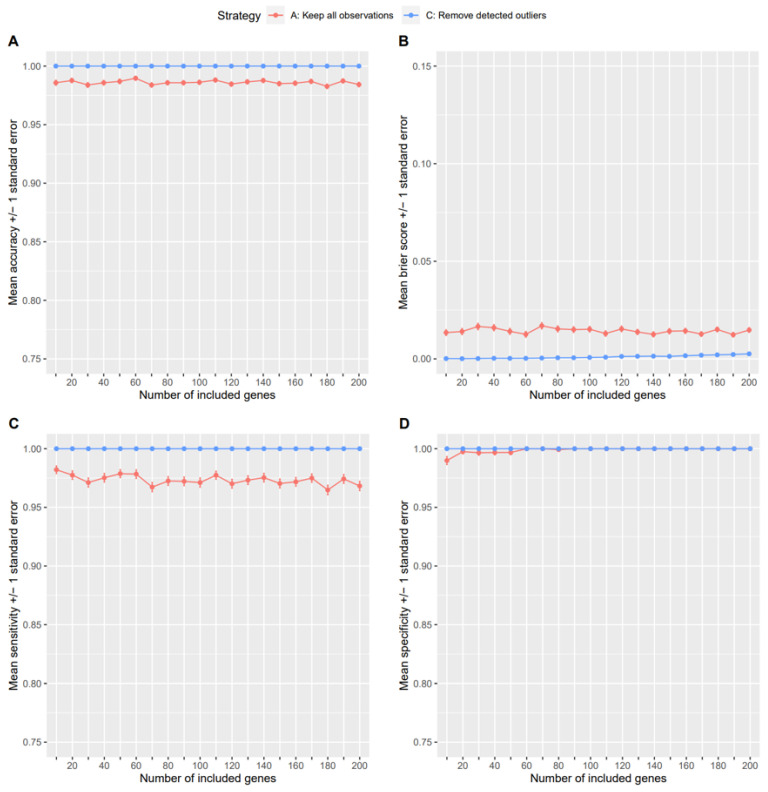
Performances for different outlier handling strategies for the WNV dataset. The figure shows mean values with error bars representing standard errors for accuracies (**A**), Brier scores (**B**), sensitivities (**C**), and specificities (**D**), respectively, when using a SVM for classification and for different numbers of most differentially expressed genes to be included in the models.

After cross-validation of the classifiers for the strategy where all samples remain in the dataset, the one outlier was removed from the WNV group accordingly and the classifier performances were re-evaluated. Figure 8 shows the classification performances for RF. Comparable results were obtained for SVM and LDA, with LDA showing overall less good performance, and can be inspected in the Supplementary Materials. The classification performances are presented as mean (+/− standard error) values for accuracy, Brier score, sensitivity, and specificity from 100 cross-validation runs in dependence of the number of genes included as predictor variables. For all four measures, strategy C, in which the detected outliers were removed, consistently showed better performances than strategy A, in which all samples are kept. However, for specificity, the two strategies performed similarly as the number of predictor variables increased. It should be noted that the classification performance did not change considerably with increasing number of genes, indicating that the inclusion of few genes already leads to a highly accurate discrimination of the groups. Especially when the outlier was removed, the highest possible correct classification rate could already be achieved with ten top differentially expressed genes as predictor variables, i.e., an accuracy of 1.0.

### 3.4. Findings for Differentially Expressed Genes Analyses

**Figure 9:**
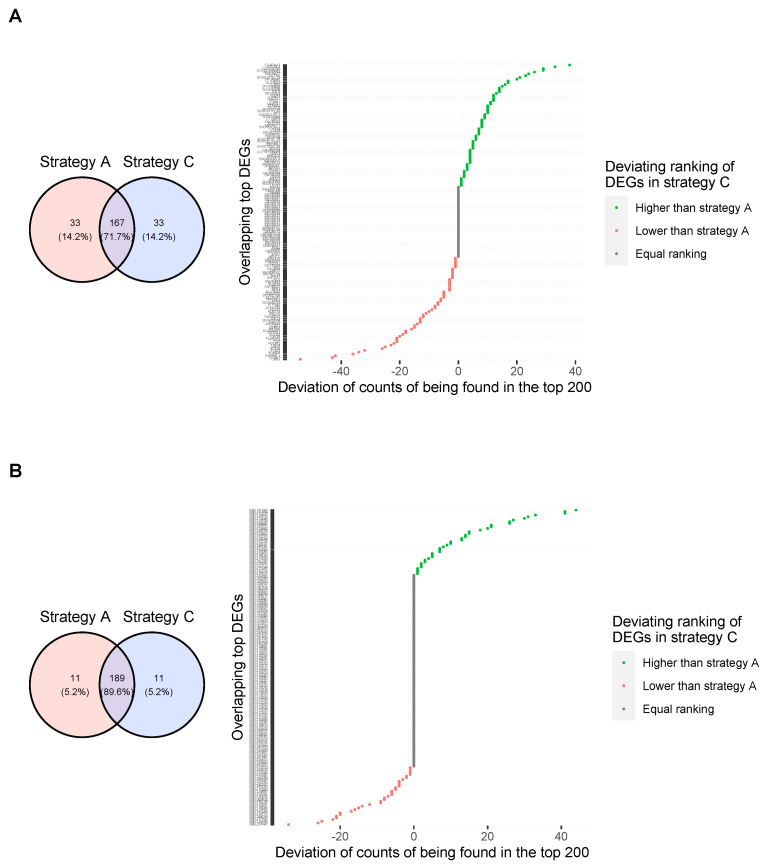
Relative and absolute overlap of the cross-validated top DE genes found in the two real-world datasets before and after outlier removal. Subfigure (**A**) shows the overlap for the top 200 DE genes before and after removing the outliers (strategy A and strategy C) for the SARS-CoV-2 dataset, as well as the deviating ranking of the DE genes. Accordingly, subfigure (**B**) presents the intersection between the top 200 genes found in the WNV dataset before and after removal of the outlier and the divergent ranks of the respective DE genes.

In addition to the effect on classifier performances for the simulated and real-world data, we were also interested in the influence of outlier removal on the differentially expression (DE) analysis, as this analysis is usually run to select predictor genes for the ML models. Therefore, we inspected the genes or transcripts that were most frequently found as belonging to the top DEGs within cross-validation and compared the findings of each strategy of outlier handling. Figure 9 shows Venn diagrams representing the overlap of cross-validated top 200 genes found by DE analysis before and after removing significant outliers for the two real-world datasets (left plots) as well as the divergence in ranking of the respective overlapping genes between the two strategies (right plots). The upper plots (A) show the intersection and diverging ranks of top 200 DE genes between strategies A (keep all samples) and C (remove significant outliers from bootstrap) for the SARS-CoV-2 dataset. The plots below (B) are structured in the same way and show the overlap and the divergent ranks for the WNV dataset, respectively.

For the SARS-CoV-2 dataset, the overlap between the top 200 DE genes of strategy A and strategy C is 71.7%. Looking only at the top 200 genes, 33 genes represented in the top 200 of one strategy are not found among the top 200 of the other strategy. Of the 167 overlapping DEGs in the respective top 200, the ranking did not change for 40 DEGs after removing the outliers. For three overlapping DEGs, the frequencies of being reported in the top 200 differed by more than 40 from those before the removal of the outliers. One gene, i.e., FAM92B, was found to be within the top100 DE genes after removal of the outliers but not even in the top 200 DE genes when all samples were included for DE analysis.

As expected, the differences were smaller in the WNV dataset, where only one outlier was removed, i.e., the overlap between the top differentially expressed transcripts between the two strategies was larger, namely, 89,6%. In addition, for more overlapping DE genes, i.e., 115, the ranking of counts being reported in the top 200 did not change after removal of the outlier in the WNV group. Three overlapping DE genes were reported in the top 200 with a frequency that differed by more than 40 after the removal of the outlier. Notably, one transcript (microarray probe identifier ILMN_1790472 for gene SLC25A28, a mitochondrial iron transporter) was found in all 100 cross-validation runs for strategy C, where the outlier was removed, to be within the top 200 differentially expressed transcripts, but for strategy A, where the outlier was not removed, this transcript was not present in the top 200 after cross-validation.

### 3.5. Run Time Evaluation

The analysis of a single dataset with our proposed approach involves *k* runs for the cross-validation, where each of these runs involves as main steps the differential expression analysis, fitting of the classifier with *m* subsets of predictor genes, as well as *B* runs to assess and test outlier probabilities. Thus, the procedure has a run time complexity of O(kmB). The run time may, however, be significantly influenced by the specific ML method and the size of the dataset.

The evaluation of one simulated dataset with 110 samples and 1000 genes, including the outlier probability estimation with 100 bootstrap runs and cross-validation of the classifier performances with and without simulated outliers and detected outliers, respectively, for different numbers of genes took less than ten minutes on a standard PC with 16 GB RAM and using one core. The bootstrap procedure for outlier probability estimation itself using bagplot and PCA-Grid took 80 s and 25 s, respectively, without parallelization.

The computational cost for the bootstrap procedure to assess outlier probabilities in the real-world datasets varied between 10 and 35 min. The individual run time depended on the used outlier detection method (bagplot or grid approach), the sample size, and the number of genes present in the data. Again, the times were obtained from analysis on a single PC with using only one core. Parallelization would, of course, decrease run times substantially.

## 4. Discussion

A large number of studies on transcriptomics classifiers has been presented in the last twenty-five years. Although many expectations were connected with these developments, transcriptomics classifiers ‘have so far not revolutionized clinical practice, and their clinical utility has not been satisfactorily established’ [41]. Nevertheless, for many diseases there is still progress and improvement in the development of transcriptomics classifiers and a chance to use such classifiers in clinical decision-making [42,43].

One obstacle between reporting a high-performant classifier and its clinical usability for diagnosis or prediction is the different composition of the populations from which training and evaluation samples were taken and the population in which the classifier is to be applied. Outliers especially can strongly bias the estimation of a classifier’s performance. Typical cross-validation procedures (‘leave-one-out’, *k*-fold, bootstrap) yield average values for accuracy and other measures of classifier performance. This also includes to some extent the effect of outliers, because some cross-validation subsets will contain or not contain outlying samples. However, a direct comparison of the classifier performance in absence or presence of outliers is not given. Therefore, we studied here in particular how incorporation or removal of outliers in the training and test datasets affect the performance of transcriptomic classifiers. This provides researchers a broader view of how a classifier can perform under extreme deviations between the distributions of the data used for training and evaluation and the distribution of data if the classifier is in practical use.

In our simulation study and in the two real-world examples, the removal of outliers changed the accuracy with different strength in the two-group and four-group scenario. The effect was smaller in the four-group scenario, where naturally sample size is larger, and consequently the effect of individual samples is lower. As we considered that the effect of outliers on the performance estimation also depends on the location of the outliers, we simulated data with more and less distant outliers. However, in our simulation examples with the two-study group, outliers were mostly located between the groups. We assume that if outliers of one group are located in the opposite direction of the second group, their removal can also decrease the performance. In the latter case, removal of outliers would decrease the separability of the two groups. In the case of four study groups, additional location of outliers occurred and a small decrease of classifier performance was observed when excluding outliers. Therefore, we want to make clear that we do not advocate in general to remove outliers, but to study their effect on the performance estimation of a transcriptomic classifier.

Using either bagplots or a grid-based method in the space of the first two principal components allows for a binary decision whether a particular sample is an outlier. This might sometimes be too strict a criterion. Therefore, we developed a new bootstrap approach to determine the outlier probability of individual samples. This new method can help analysts to train models on different subsets of the data based on different thresholds for the outlier probability. In addition, the outlier probabilities can help interdisciplinary teams of bioinformaticians and clinical partners detect and discuss the relevance of extreme samples in the training data.

One step during classifier training is the selection of a transcriptomic finger print in the form of genes or transcripts used as predictor variables. The subsets of selected genes change, of course, when keeping or removing outliers. Thus, the biological interpretation of a transcriptomic finger print can change, too. In Section 3.4, we demonstrated this effect for single genes that extremely changed their rank after outlier removal. For a transcriptomic classifier, it is often desirable to also understand the biological role of the selected genes, for example, by means of gene-set enrichment analysis. In 2003, Pepe et al. showed how to use bootstrap to derive the distribution of ranking lists of differentially expressed genes [44]. Our method to determine individual outlier probabilities allows for extending this idea and deriving the distribution of such ranking lists for data with and without outliers.

## 5. Conclusions

Very often, a divergence can be observed between the performance of a transcriptomic classifier found in the evaluation by cross-validation and the performance of the model found when being applied on independent test sets. We demonstrated that outliers in either the training or test data can seriously change the separability of transcriptomic data from different groups, and thus the performance estimates obtained. We further presented a new approach of estimating a classifier’s performance by detecting and removing outliers in the high-dimensional training and test data. Whereas outlier detection in the space of the first two principal components by bagplots or a grid-approach is a solely binary decision (a sample either flagged as an outlier or not), we present a bootstrap approach to also determine the outlier probability of each particular sample. This enables us to evaluate the performance of their models by including or excluding outliers based on these probabilities. In summary, our approach can help to assess a classifier’s performance with a higher robustness and to avoid a too optimistic estimate that later turns out as not tenable.

## Figures and Tables

**Table 1:** Structure of the three different datasets that were used for assessment of outlier probabilities and classifier models. The SARS-CoV-2 dataset was generated by RNA-Seq, and the WNV dataset was generated by DNA microarrays. Abbreviations: ARI: acute respiratory illness; PBMC: peripheral blood mononuclear cell; WNV: West Nile Virus.

Dataset	Number of Samples	Number of Transcripts	Study Design	
Simulated dataset	N=110	*D* = 1000	Group 1:	55 samples including 5 outliers
			Group 2:	55 samples including 5 outliers
Subset of SARS-CoV-2 study (GSE163151)	N=258	*D* = 26,485	Group ‘COVID’:	Nasopharyngeal swabs from 138 humans with ARI caused by SARS-CoV-2
			Group ‘Other virus’:	Nasopharyngeal swabs from 120 humans with ARI caused by other viruses
Subset of WNV study (GSE46681)	N=78	*D* = 47,323	Group ‘Mock’:	PBMCs from 39 humans incubated in medium alone
			Group ‘WNV’:	PBMCs from 39 humans infected with WNV ex vivo

**Table 2:** Twenty samples most frequently detected as outliers in the SARS-CoV-2 dataset during bootstrap–bagplot analysis. In addition to sample ID, the number of occurrences in 100 bootstrap runs and the number of bootstrap runs the sample was detected as outlier are presented. Furthermore, the estimated outlier probability with a 95%-confidence interval and the *p*-value from the binomial test are presented.

Sample ID	Occurrences in Bootstrapping	Detected as Outlier	Outlier Probability	95%-CI	*p*-Value
GSM4972835	68	68	1.00	[0.96, 1.00]	<0.001
GSM4972888	64	64	1.00	[0.95, 1.00]	<0.001
GSM4972929	68	68	1.00	[0.96, 1.00]	<0.001
GSM4972877	67	66	0.99	[0.93, 1.00]	<0.001
GSM4972927	66	65	0.98	[0.93, 1.00]	<0.001
GSM4972944	66	63	0.95	[0.89, 1.00]	<0.001
GSM4973094	60	57	0.95	[0.88, 1.00]	<0.001
GSM4972878	63	59	0.94	[0.86, 1.00]	<0.001
GSM4973123	57	53	0.93	[0.85, 1.00]	<0.001
GSM4972907	63	45	0.71	[0.61, 1.00]	<0.001
GSM4973051	61	43	0.70	[0.59, 1.00]	0.001
GSM4972892	87	59	0.68	[0.59, 1.00]	0.001
GSM4972872	72	43	0.60	[0.49, 1.00]	0.062
GSM4972919	66	38	0.58	[0.47, 1.00]	0.134
GSM4972891	58	33	0.57	[0.45, 1.00]	0.179
GSM4972875	67	37	0.55	[0.44, 1.00]	0.232
GSM4972971	59	31	0.53	[0.41, 1.00]	0.397
GSM4972973	66	33	0.50	[0.39, 1.00]	0.549
GSM4972946	60	29	0.48	[0.37, 1.00]	0.651

**Table 3:** Ten samples most frequently detected as outliers in the WNV dataset during bootstrap–bagplot analysis. The table includes sample ID, the number of occurrences in 100 bootstrap runs, and the number of bootstrap runs where the sample was detected as outlier. Additionally, the estimated outlier probability with a 95%-confidence interval and the *p*-value from the binomial test are presented.

Sample ID	Occurrences in Bootstrapping	Detected as Outlier	Outlier Probability	95%-CI	*p*-Value
GSM1133935	71	55	0.77	[0.68, 1.00]	<0.001
GSM1058065	66	23	0.35	[0.25, 1.00]	0.995
GSM1133954	68	21	0.31	[0.22, 1.00]	1.00
GSM1058069	69	18	0.26	[0.18, 1.00]	1.00
GSM1133940	62	12	0.19	[0.12, 1.00]	1.00
GSM1058085	62	11	0.18	[0.10, 1.00]	1.00
GSM1058062	64	11	0.17	[0.10, 1.00]	1.00
GSM1133950	62	10	0.16	[0.09, 1.00]	1.00
GSM1058064	61	9	0.15	[0.08, 1.00]	1.00
GSM1058082	64	8	0.12	[0.06, 1.00]	1.00
GSM1133931	64	8	0.12	[0.06, 1.00]	1.00

## Data Availability

The data presented in this study are openly available in Gene Expression Omnibus Archive (https://www.ncbi.nlm.gov/geo (accessed 1 February 2022)) under the accession numbers GSE163151 and GSE46681. No new data were created or analyzed in this study. Data sharing is not applicable to this article.

## Supplementary Materials

The following supporting information can be downloaded at: https://www.mdpi.com/article/10.3390/genes14020387/s1, Figure S1: Boxplots of accuracies and Brier scores for simulation data using SVM in a two-study-group scenario; Figure S2: Boxplots of sensitivities and specificities for simulation data using SVM in a two-study-group scenario; Figure S3: Boxplots of positive and negative predictive values for simulation data using SVM in a two-study-group scenario; Figure S4: Boxplots of accuracies and Brier scores for simulation data using RF in a two-study-group scenario; Figure S5: Boxplots of sensitivities and specificities for simulation data using RF in a two-study-group scenario; Figure S6: Boxplots of positive and negative predictive values for simulation data using RF in a two-study-group scenario; Figure S7: Boxplots of accuracies and Brier scores for simulation data using LDA in a two-study-group scenario; Figure S8: Boxplots of sensitivities and specificities for simulation data using LDA in a two-study-group scenario; Figure S9: Boxplots of positive and negative predictive values for simulation data using LDA in a two-study-group scenario; Figure S10: Comparison of outlier detection methods within bootstrap approach—Boxplots of accuracies and Brier scores using SVM in a two-study-group scenario; Figure S11: Comparison of outlier detection methods within bootstrap approach—Boxplots of sensitivities and specificities using SVM in a two-study-group scenario; Figure S12: Comparison of outlier detection methods within bootstrap approach—Boxplots of positive and negative predictive values using SVM in a two-study-group scenario; Figure S13: Boxplots of accuracies and Brier scores for simulation data using SVM in a four-study-group scenario; Figure S14: Comparison of outlier detection methods within bootstrap approach—Boxplots of accuracies and Brier scores for simulation data using SVM in a four-study-group scenario; Figure S15: Mean accuracies and Brier scores (+/− 1 standard error) for SARS-CoV-2 data using SVM; Figure S16: Mean sensitivities and specificities (+/− 1 standard error) for SARS-CoV-2 data using SVM; Figure S17: Mean positive and negative predictive values (+/− 1 standard error) for SARS-CoV-2 data using SVM; Figure S18: Mean accuracies and Brier scores (+/− 1 standard error) for SARS-CoV-2 data using RF; Figure S19: Mean sensitivities and specificities (+/− 1 standard error) for SARS-CoV-2 data using RF; Figure S20: Mean positive and negative predictive values (+/− 1 standard error) for SARS-CoV-2 data using RF; Figure S21: Mean accuracies and Brier scores (+/− 1 standard error) for SARS-CoV-2 data using LDA; Figure S22: Mean sensitivities and specificities (+/− 1 standard error) for SARS-CoV-2 data using LDA; Figure S23: Mean negative and positive predictive values (+/− 1 standard error) for SARS-CoV-2 data using LDA; Figure S24: Mean accuracies and Brier scores (+/− 1 standard error) for WNV data using SVM; Figure S25: Mean sensitivities and specificities (+/− 1 standard error) for WNV data using SVM; Figure S26: Mean positive and negative predictive values (+/− 1 standard error) for WNV data using SVM; Figure S27: Figure S28: Mean sensitivities and specificities (+/− 1 standard error) for WNV data using RF; Figure S29: Mean positive and negative predictive values (+/− 1 standard error) for WNV data using RF; Figure S30: Mean accuracies and Brier scores (+/− 1 standard error) for WNV data using LDA; Figure S31: Mean sensitivities and specificities (+/− 1 standard error) for WNV data using LDA; Figure S32: Mean negative and positive predictive values (+/− 1 standard error) for WNV data using LDA; Table S1: 30 samples most frequently detected as outliers in the SARS-CoV-2 data set during bootstrap analysis using PCA-Grid; Table S2: 20 samples most frequently detected as outliers in the WNV data set during bootstrap analysis using PCA-Grid.

## Abbreviations

The following abbreviations are used in this manuscript:

ARI	Acute respiratory illness
DE	Differential expression
GEO	Gene Expression Omnibus
LDA	Linear discriminant analysis
ML	Machine learning
PBMC	Peripheral blood mononuclear cell
PCA	Principal component analysis
RF	Random forest
SVM	Support vector machine
WNV	West Nile virus
