# Supplementary material for: Assessing Outlier Probabilities in Transcriptomics Data When Evaluating a Classifier
Source: Genes (Basel). 2023 Feb 1;14(2):387. doi: 10.3390/genes14020387 (PMC9956321; doi:10.3390/genes14020387)
Supplement: Supplementary file 1 [file genes-14-00387-s001.zip › Supplementary_Figures_and_Tables.pptx]

## Slide 1
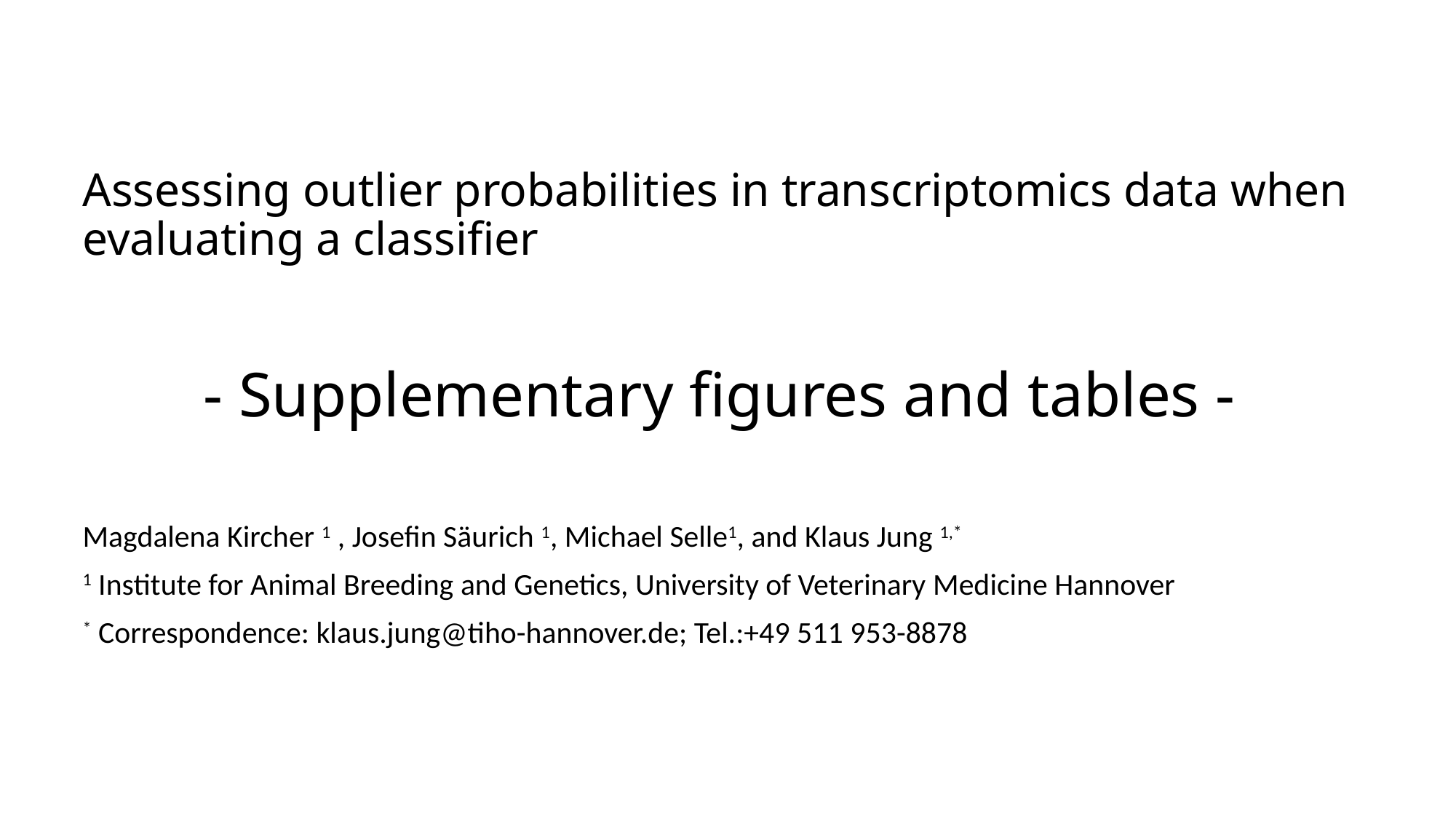

# Assessing outlier probabilities in transcriptomics data when evaluating a classifier
- Supplementary figures and tables -
Magdalena Kircher 1 , Josefin Säurich 1, Michael Selle1, and Klaus Jung 1,*
1 Institute for Animal Breeding and Genetics, University of Veterinary Medicine Hannover
* Correspondence: klaus.jung@tiho-hannover.de; Tel.:+49 511 953-8878

## Slide 2
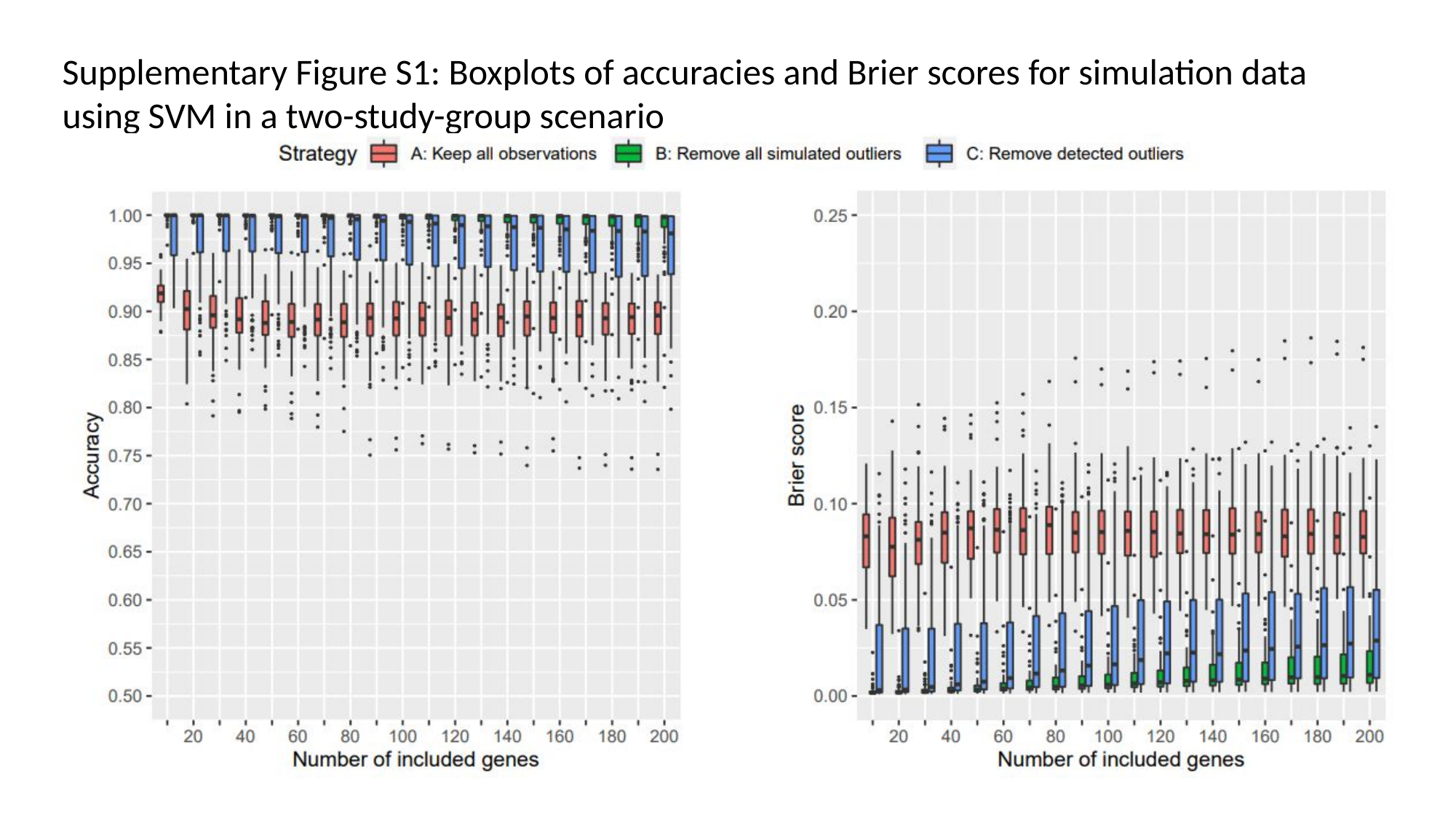

Supplementary Figure S1: Boxplots of accuracies and Brier scores for simulation data using SVM in a two-study-group scenario

## Slide 3
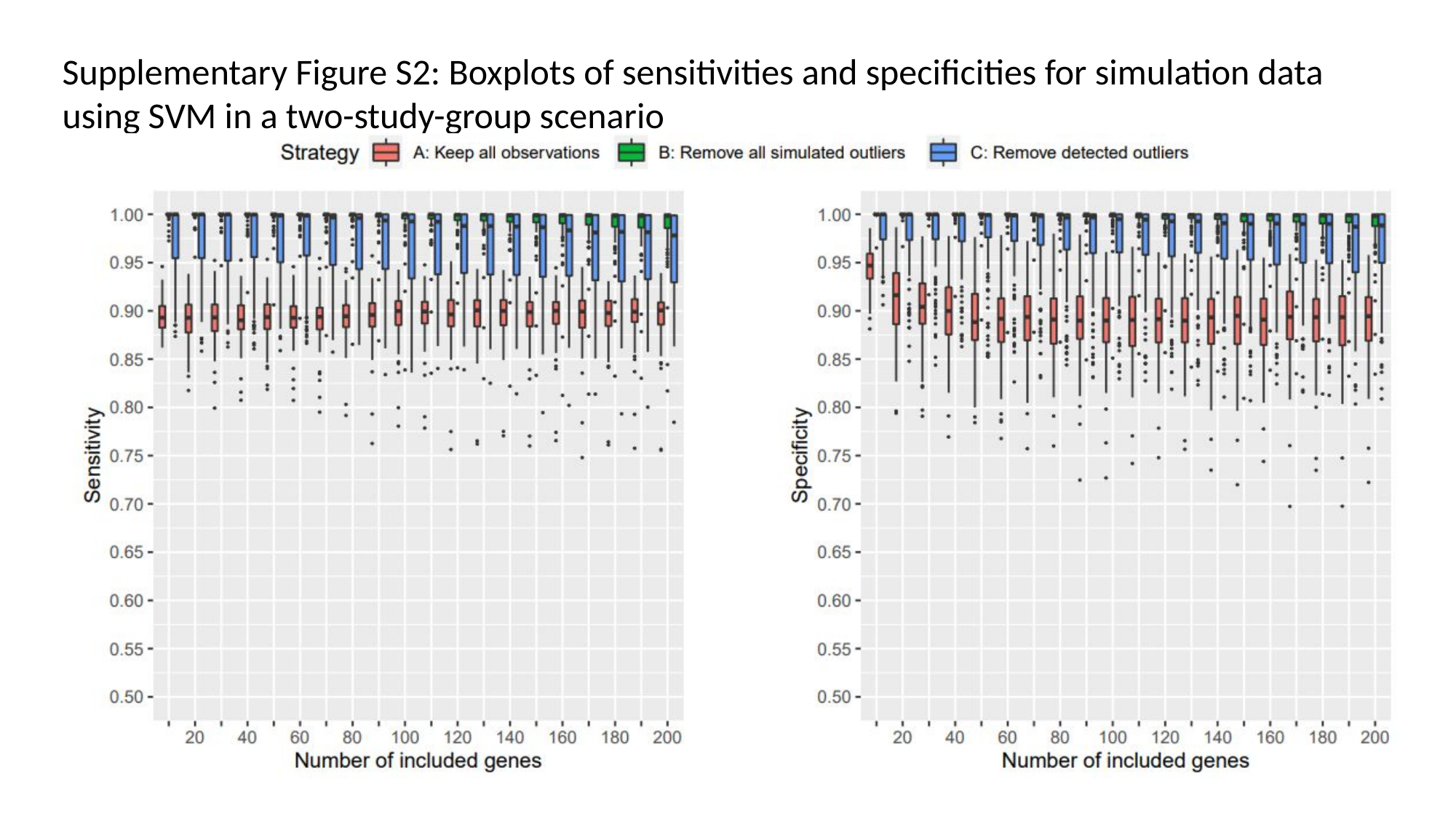

Supplementary Figure S2: Boxplots of sensitivities and specificities for simulation data using SVM in a two-study-group scenario

## Slide 4
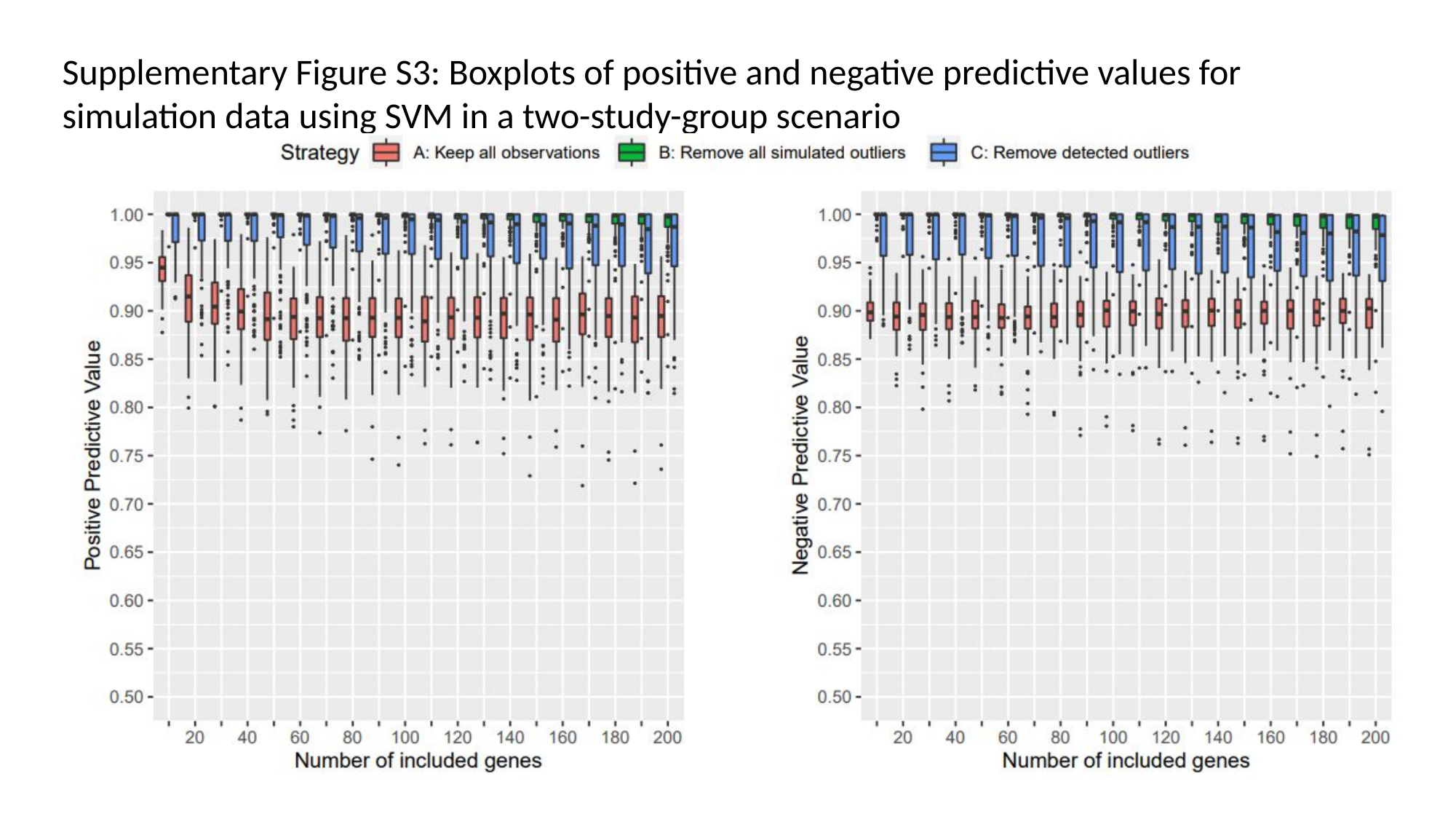

Supplementary Figure S3: Boxplots of positive and negative predictive values for simulation data using SVM in a two-study-group scenario

## Slide 5
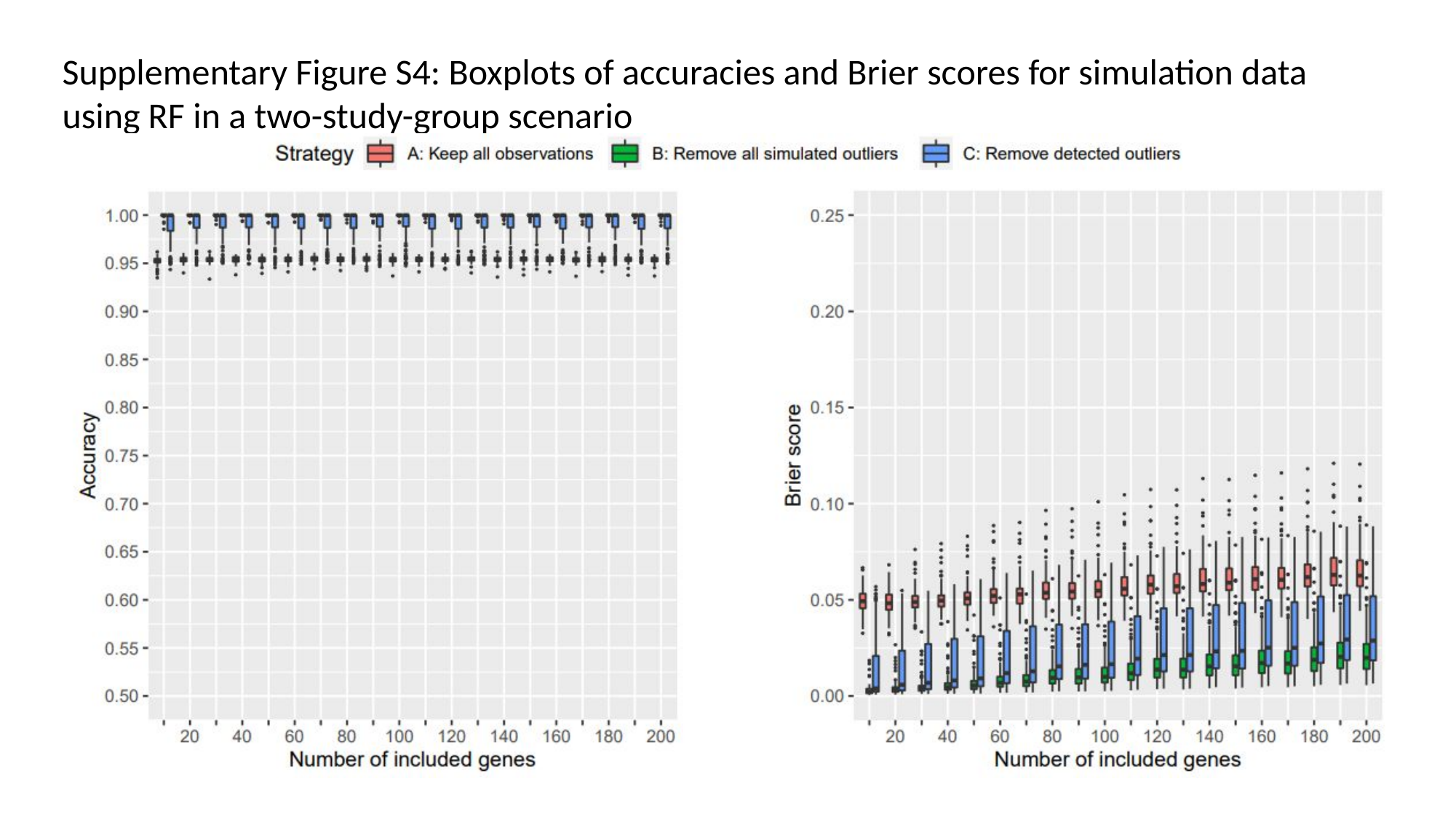

Supplementary Figure S4: Boxplots of accuracies and Brier scores for simulation data using RF in a two-study-group scenario

## Slide 6
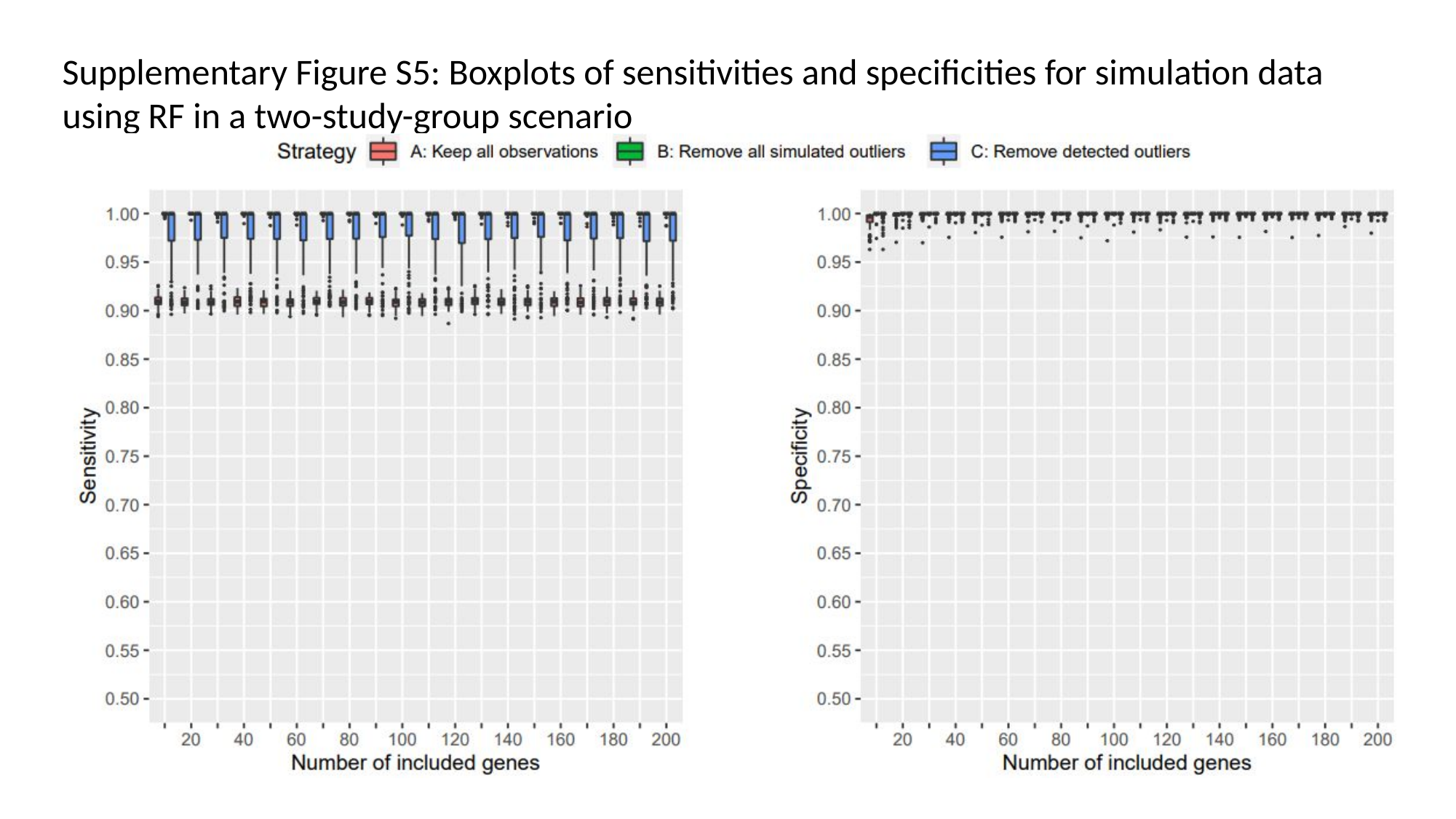

Supplementary Figure S5: Boxplots of sensitivities and specificities for simulation data using RF in a two-study-group scenario

## Slide 7
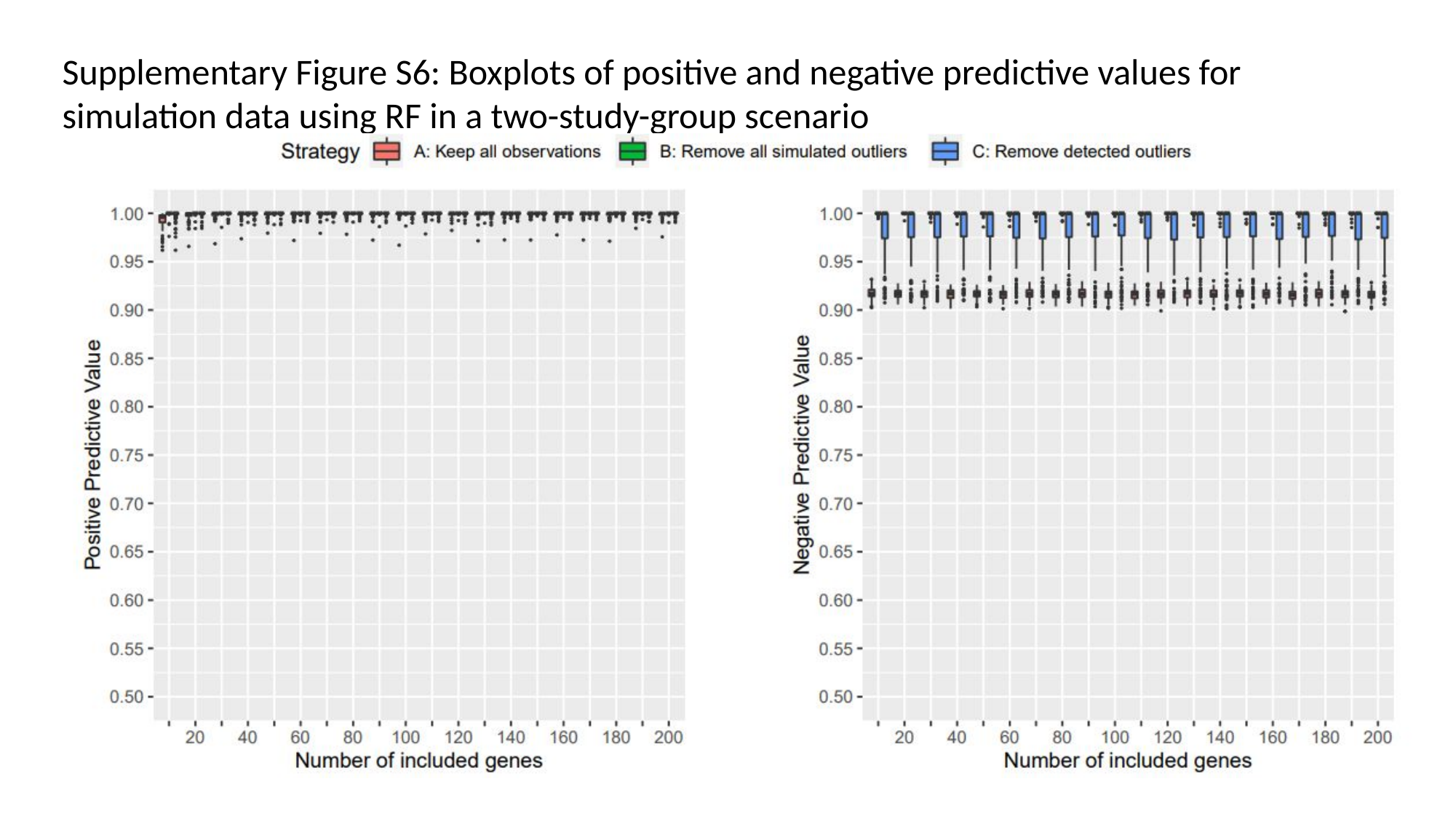

Supplementary Figure S6: Boxplots of positive and negative predictive values for simulation data using RF in a two-study-group scenario

## Slide 8
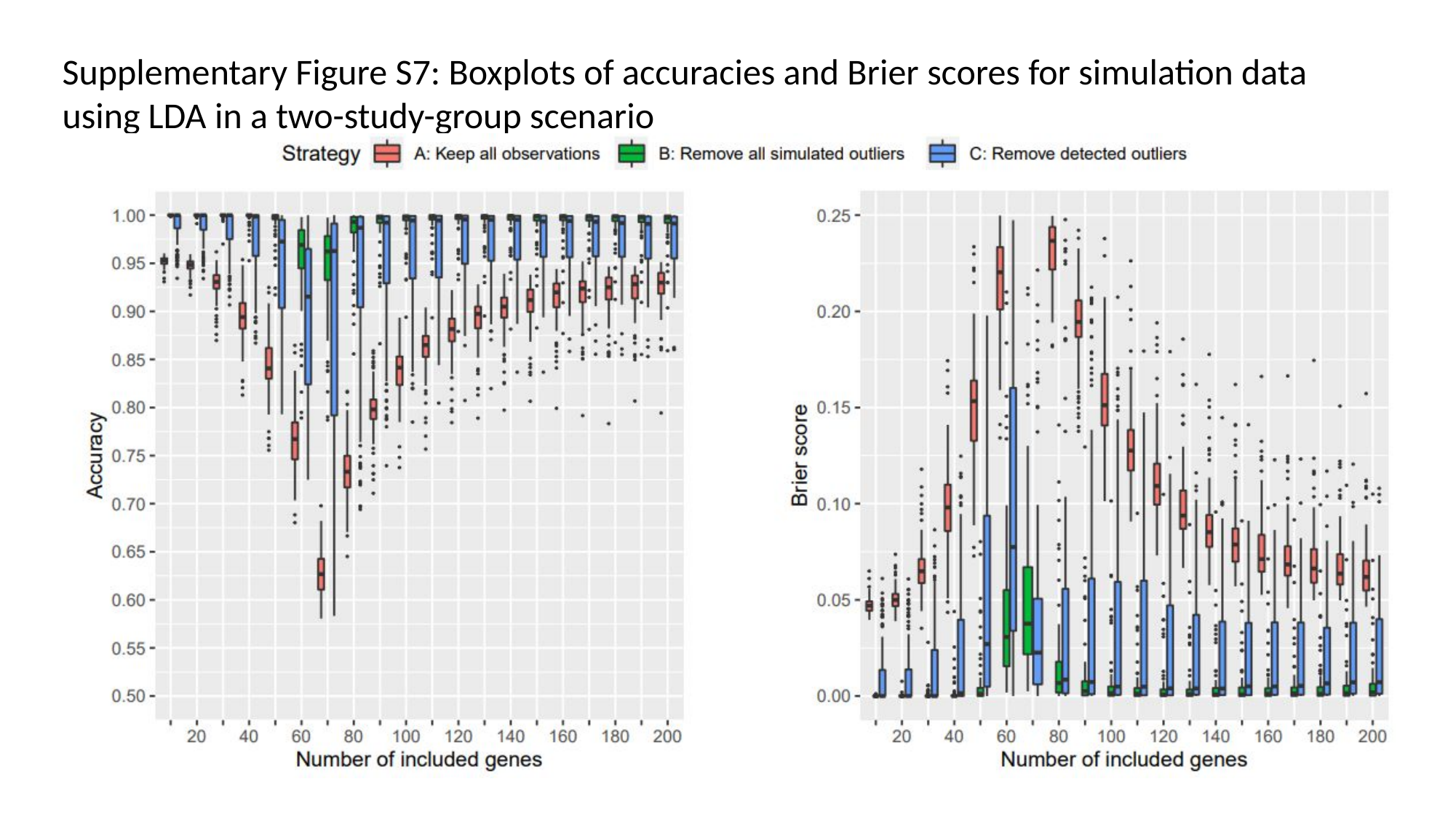

Supplementary Figure S7: Boxplots of accuracies and Brier scores for simulation data using LDA in a two-study-group scenario

## Slide 9
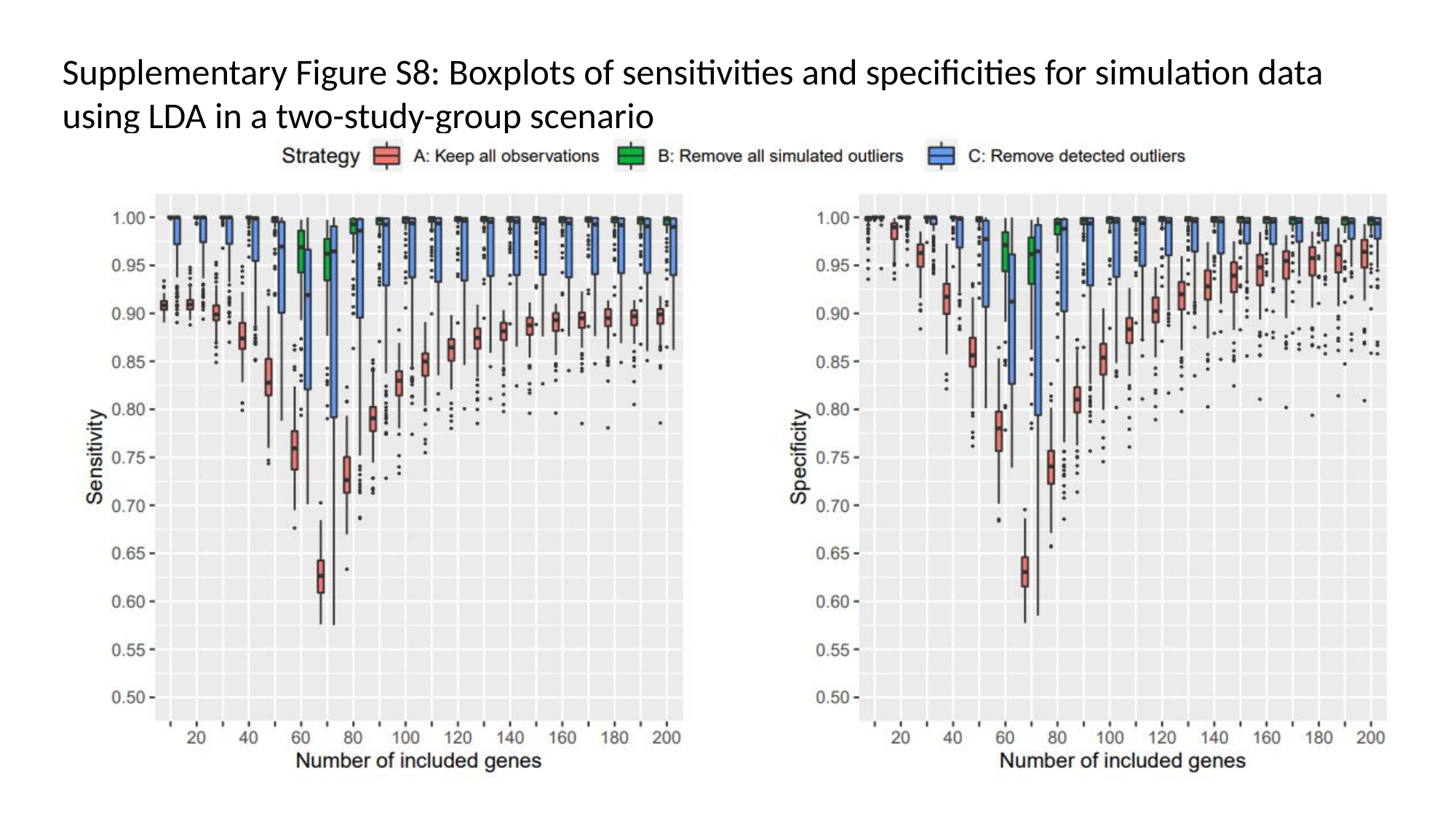

Supplementary Figure S8: Boxplots of sensitivities and specificities for simulation data using LDA in a two-study-group scenario

## Slide 10
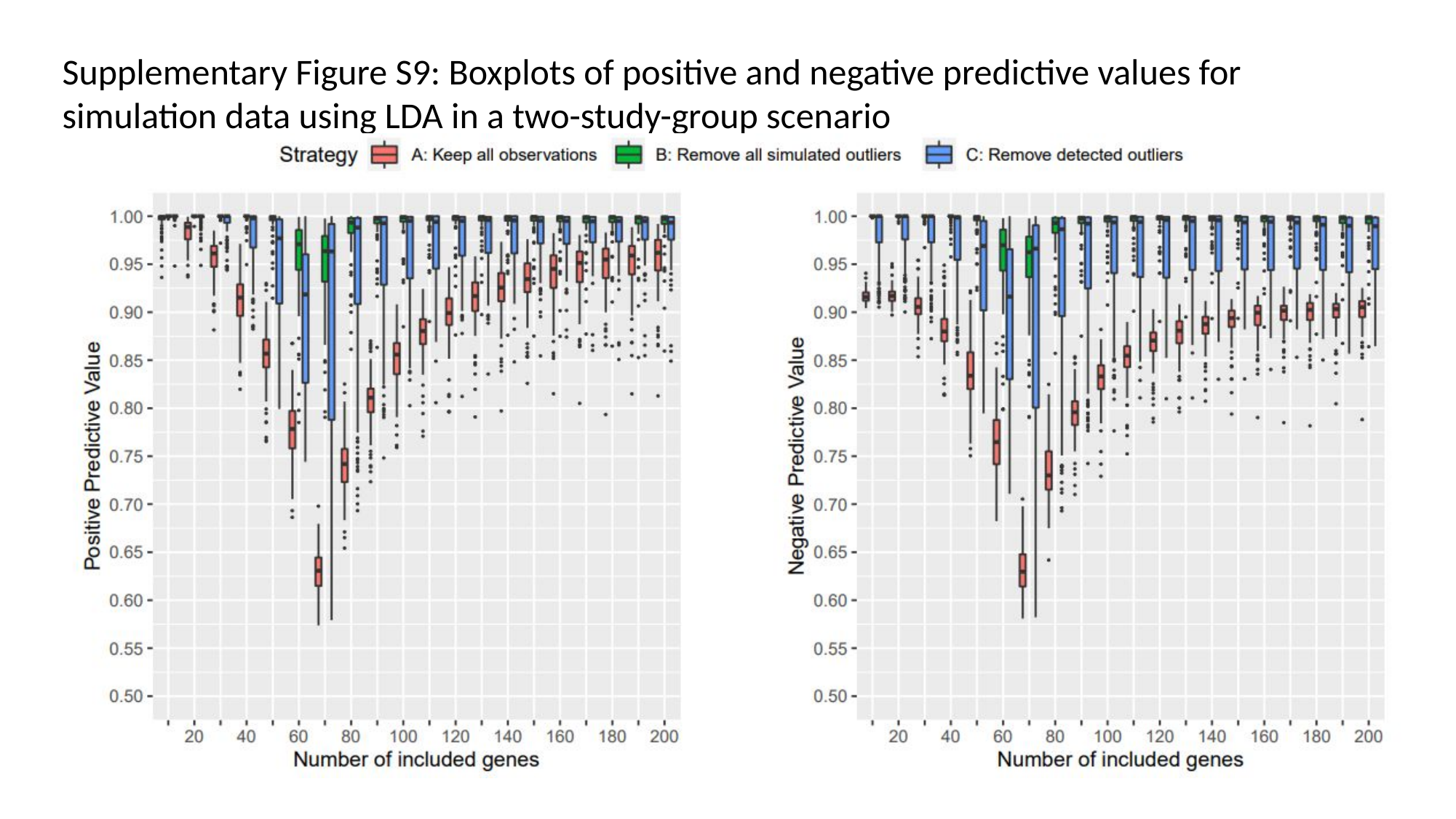

Supplementary Figure S9: Boxplots of positive and negative predictive values for simulation data using LDA in a two-study-group scenario

## Slide 11
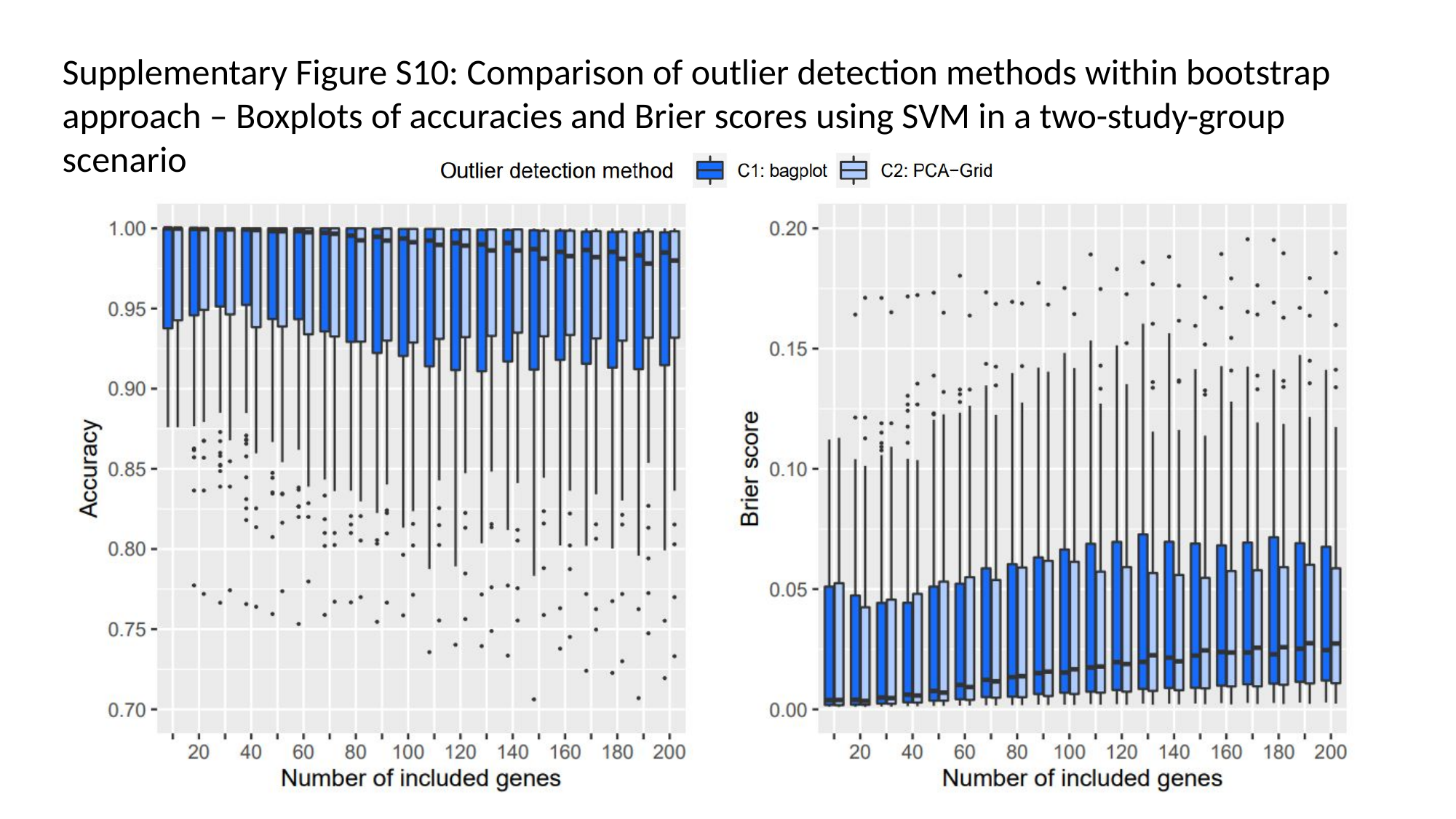

Supplementary Figure S10: Comparison of outlier detection methods within bootstrap approach – Boxplots of accuracies and Brier scores using SVM in a two-study-group scenario

## Slide 12
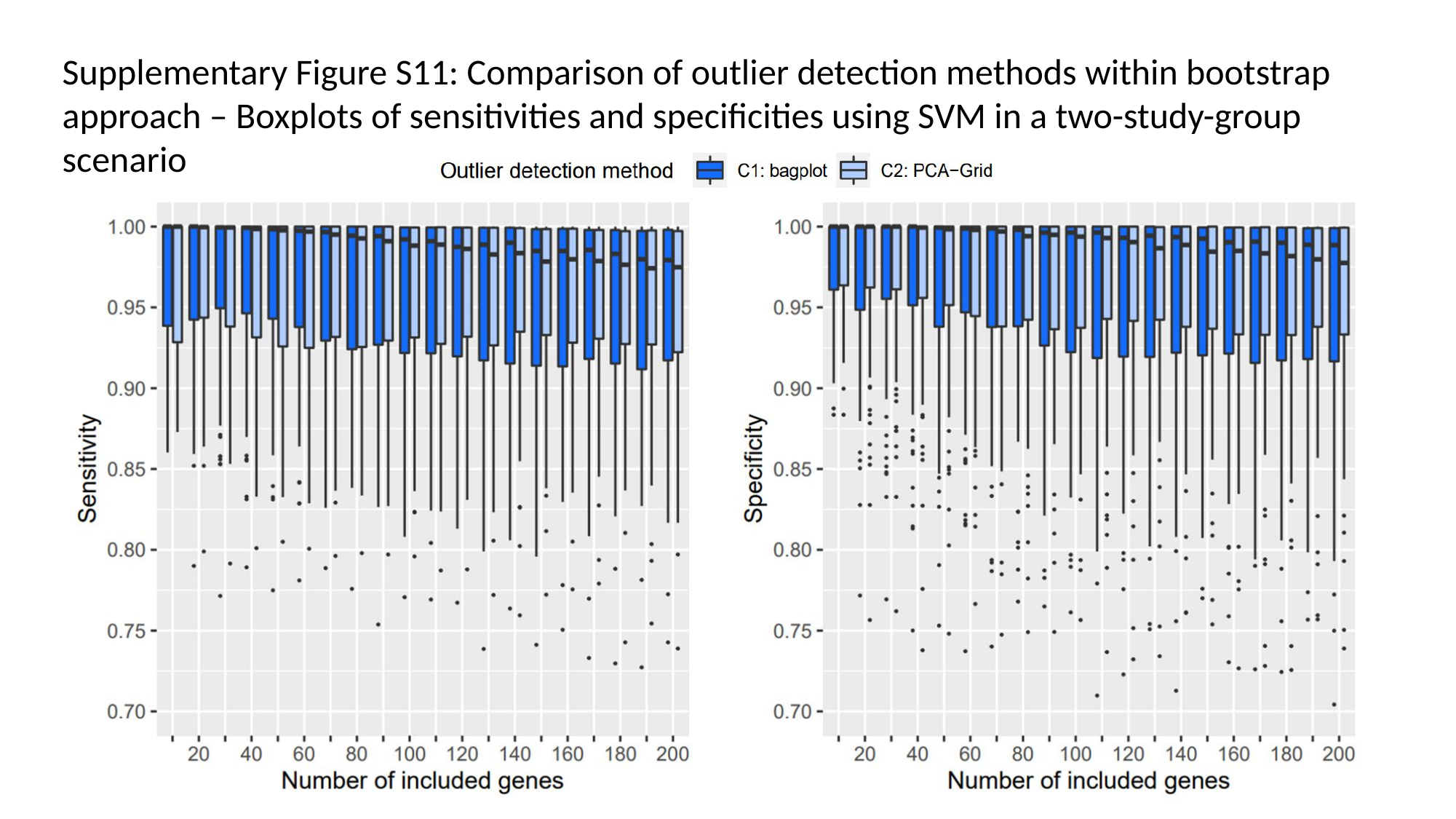

Supplementary Figure S11: Comparison of outlier detection methods within bootstrap approach – Boxplots of sensitivities and specificities using SVM in a two-study-group scenario

## Slide 13
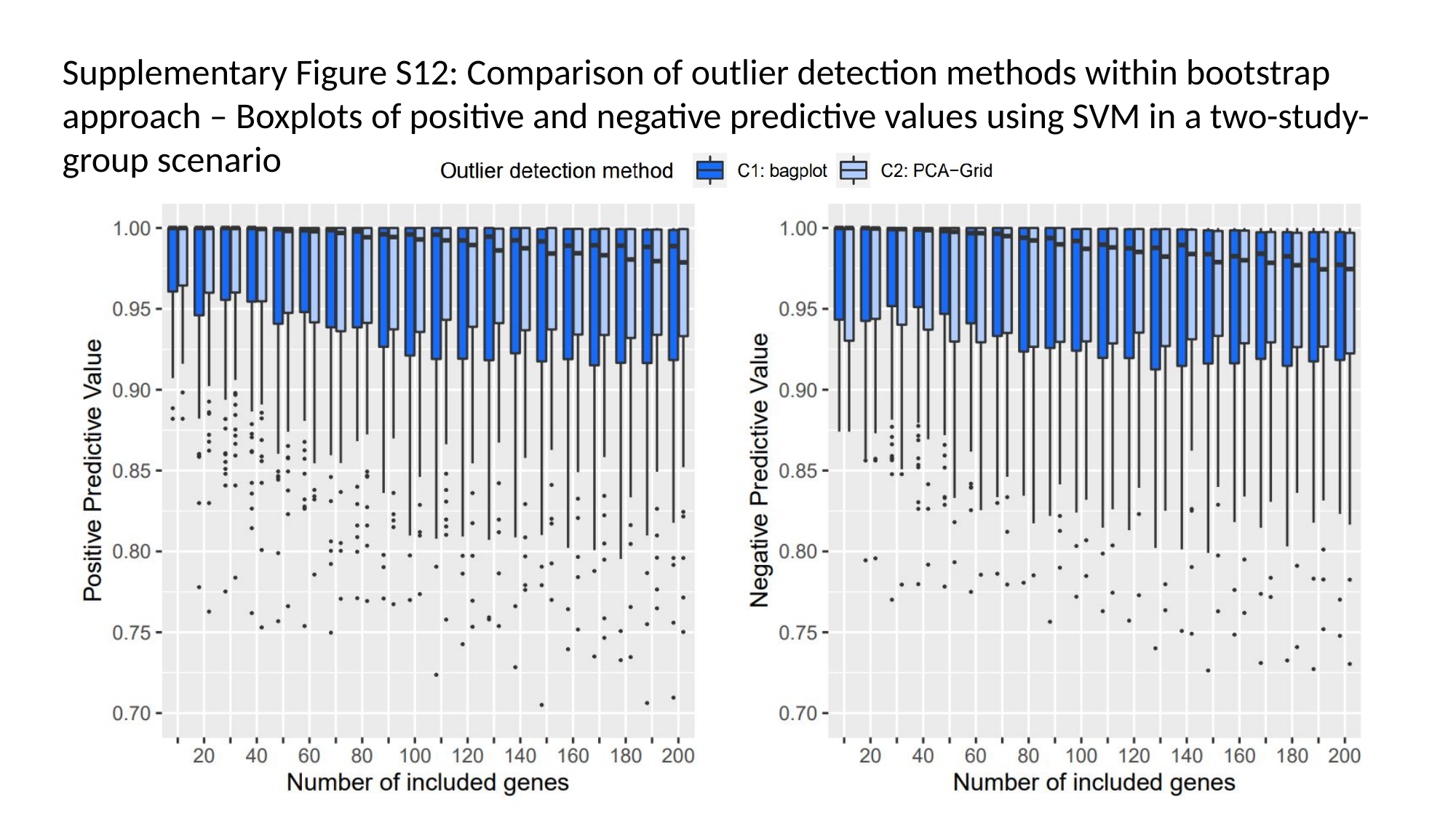

Supplementary Figure S12: Comparison of outlier detection methods within bootstrap approach – Boxplots of positive and negative predictive values using SVM in a two-study-group scenario

## Slide 14
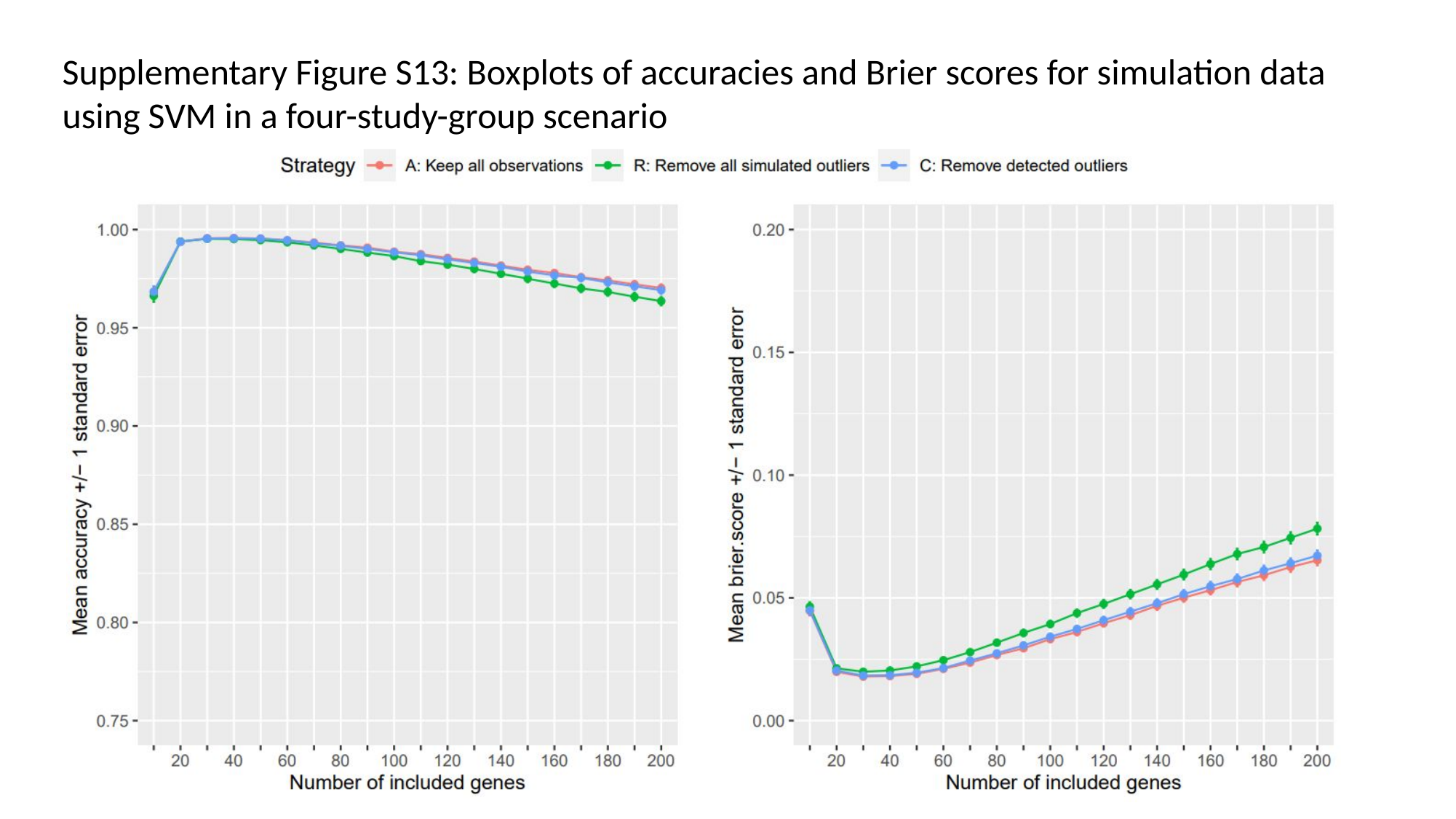

Supplementary Figure S13: Boxplots of accuracies and Brier scores for simulation data using SVM in a four-study-group scenario

## Slide 15
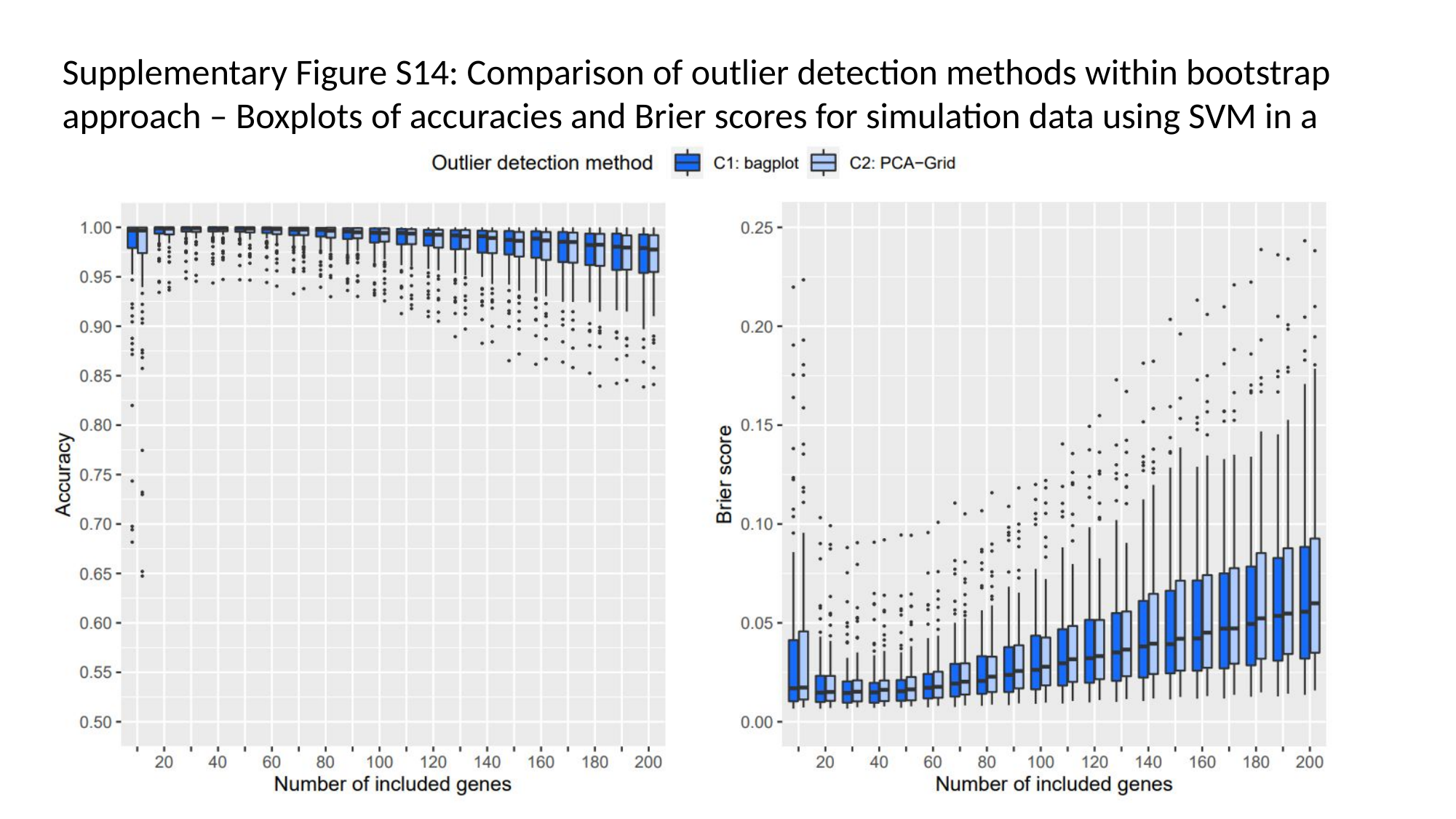

Supplementary Figure S14: Comparison of outlier detection methods within bootstrap approach – Boxplots of accuracies and Brier scores for simulation data using SVM in a four-study-group scenario

## Slide 16
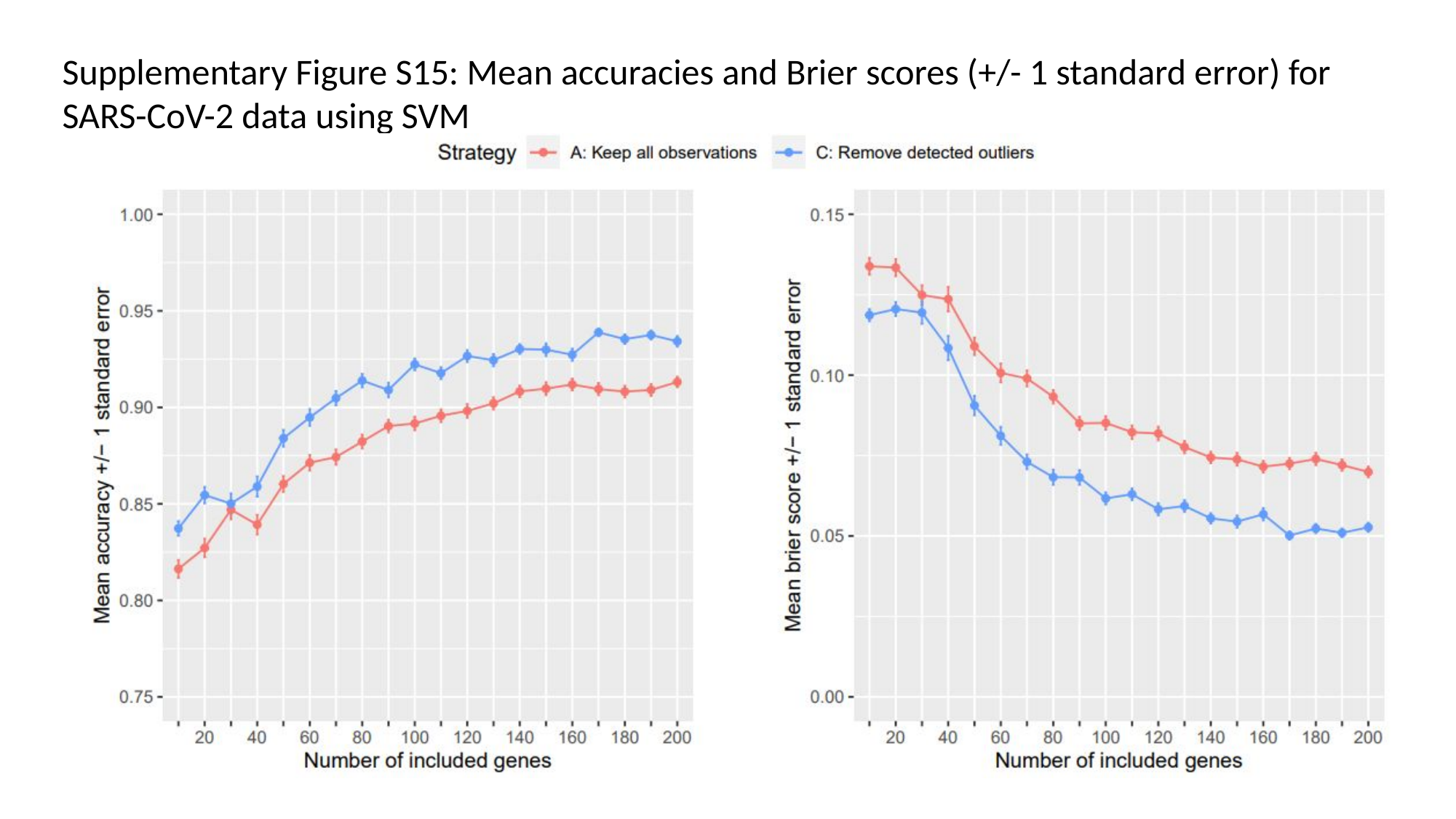

Supplementary Figure S15: Mean accuracies and Brier scores (+/- 1 standard error) for SARS-CoV-2 data using SVM

## Slide 17
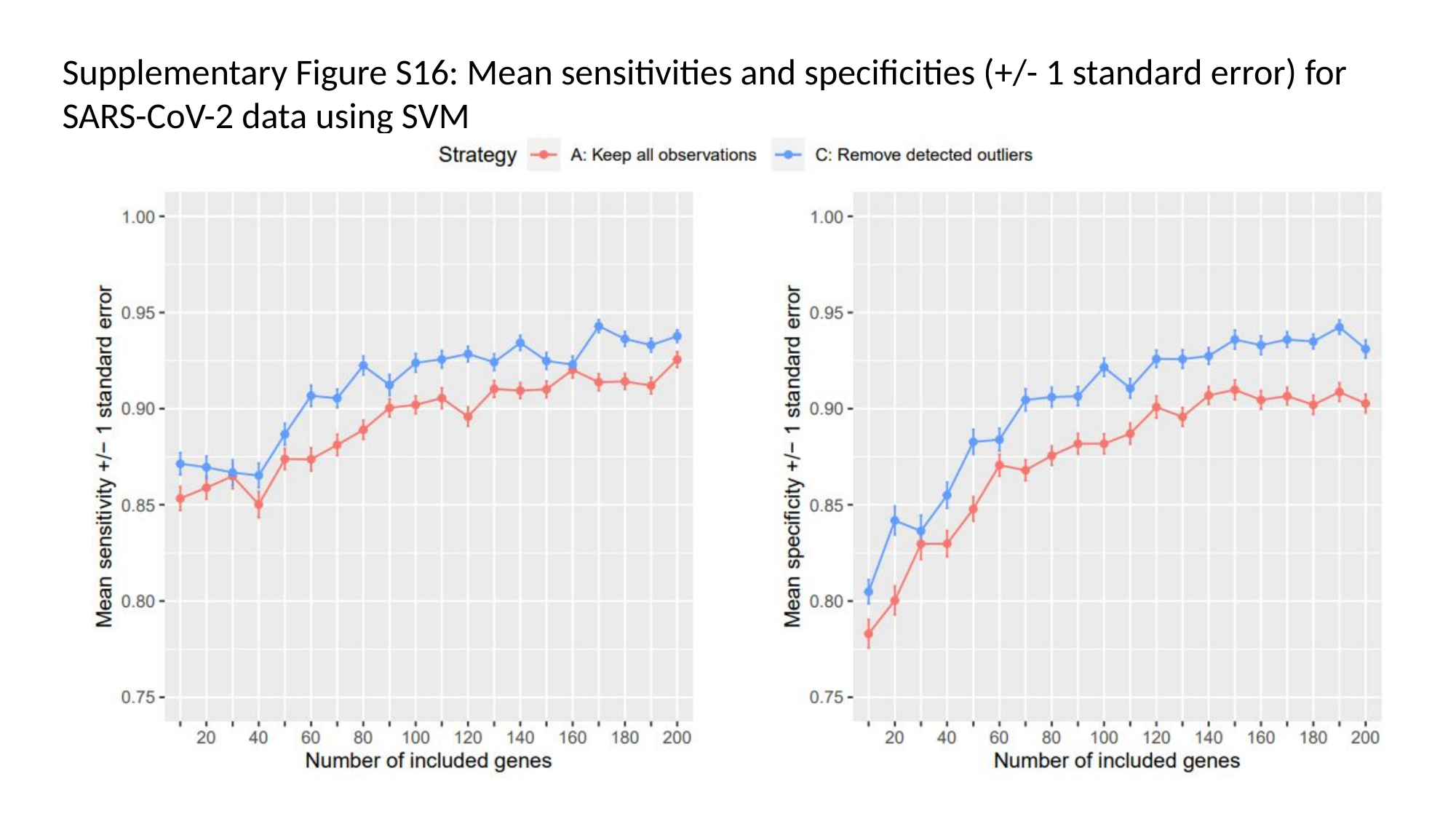

Supplementary Figure S16: Mean sensitivities and specificities (+/- 1 standard error) for SARS-CoV-2 data using SVM

## Slide 18
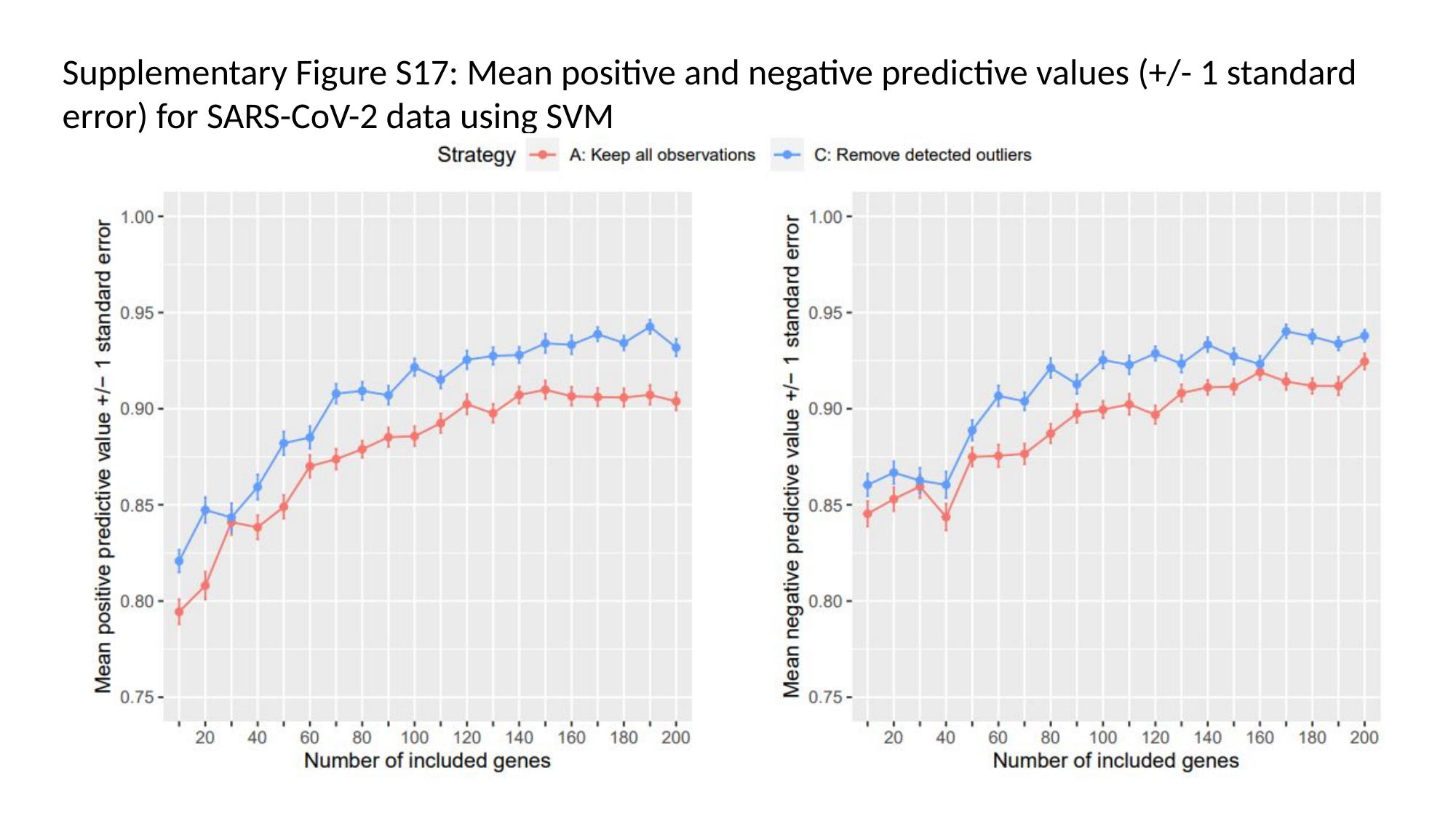

Supplementary Figure S17: Mean positive and negative predictive values (+/- 1 standard error) for SARS-CoV-2 data using SVM

## Slide 19
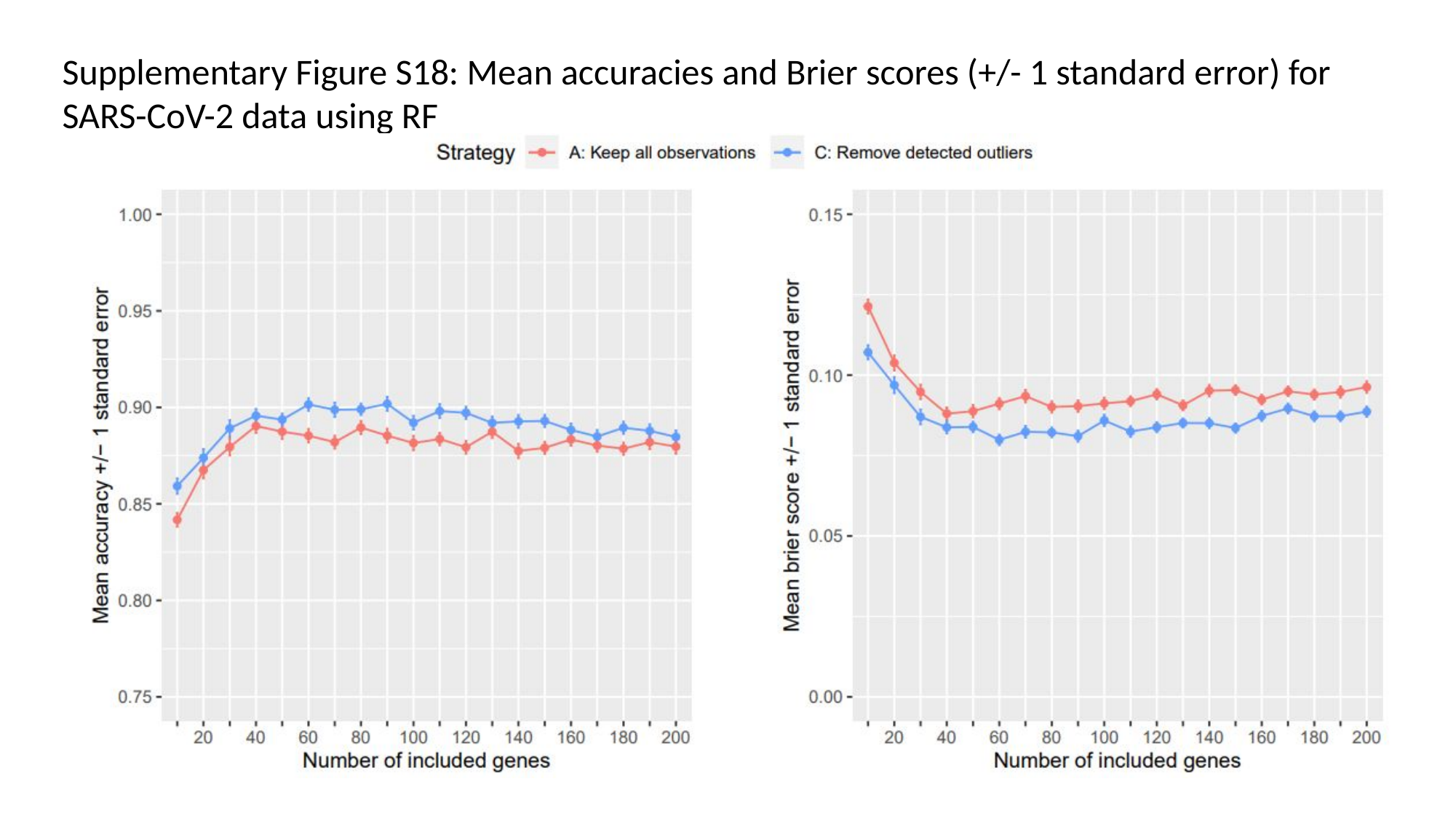

Supplementary Figure S18: Mean accuracies and Brier scores (+/- 1 standard error) for SARS-CoV-2 data using RF

## Slide 20
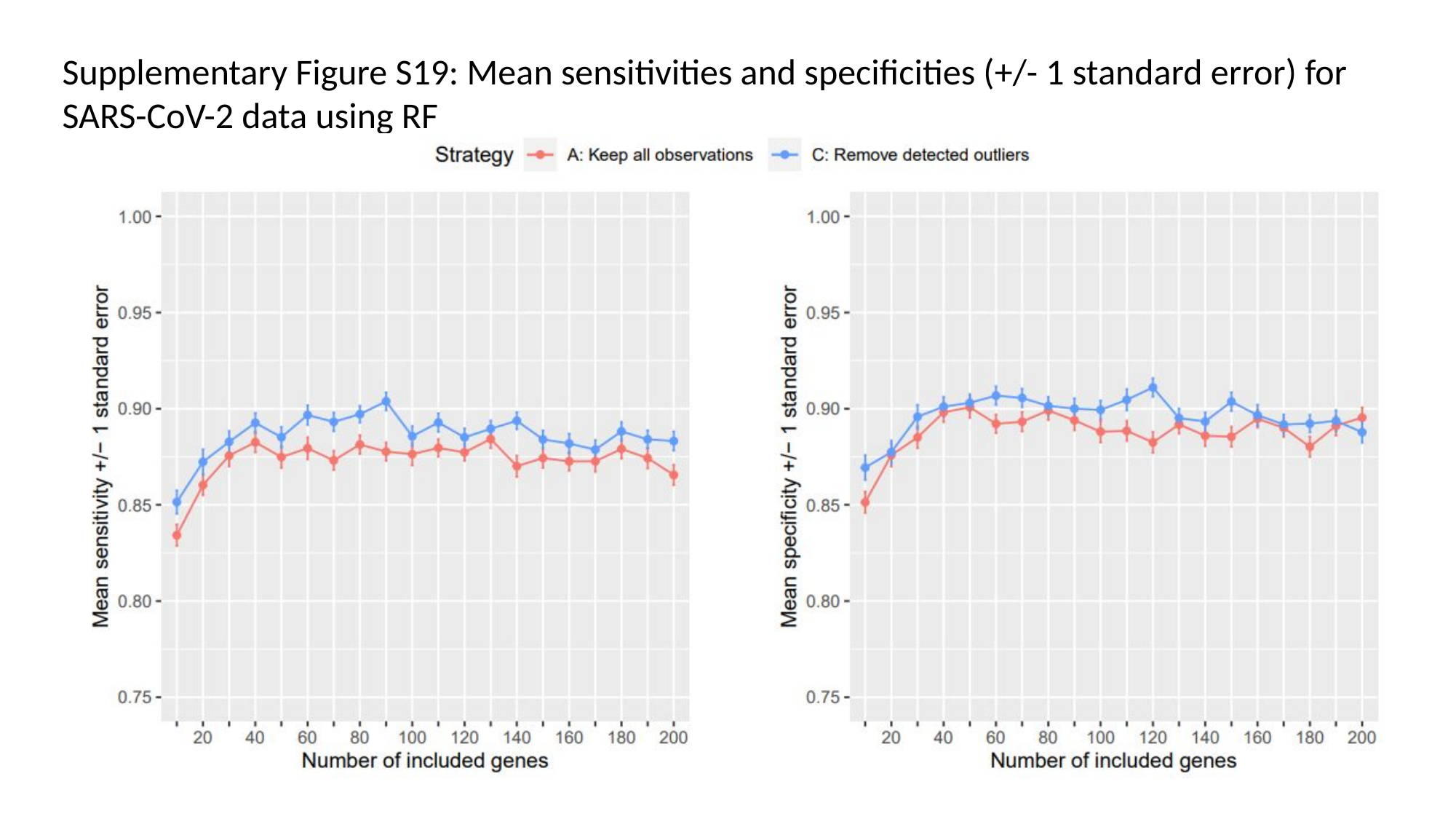

Supplementary Figure S19: Mean sensitivities and specificities (+/- 1 standard error) for SARS-CoV-2 data using RF

## Slide 21
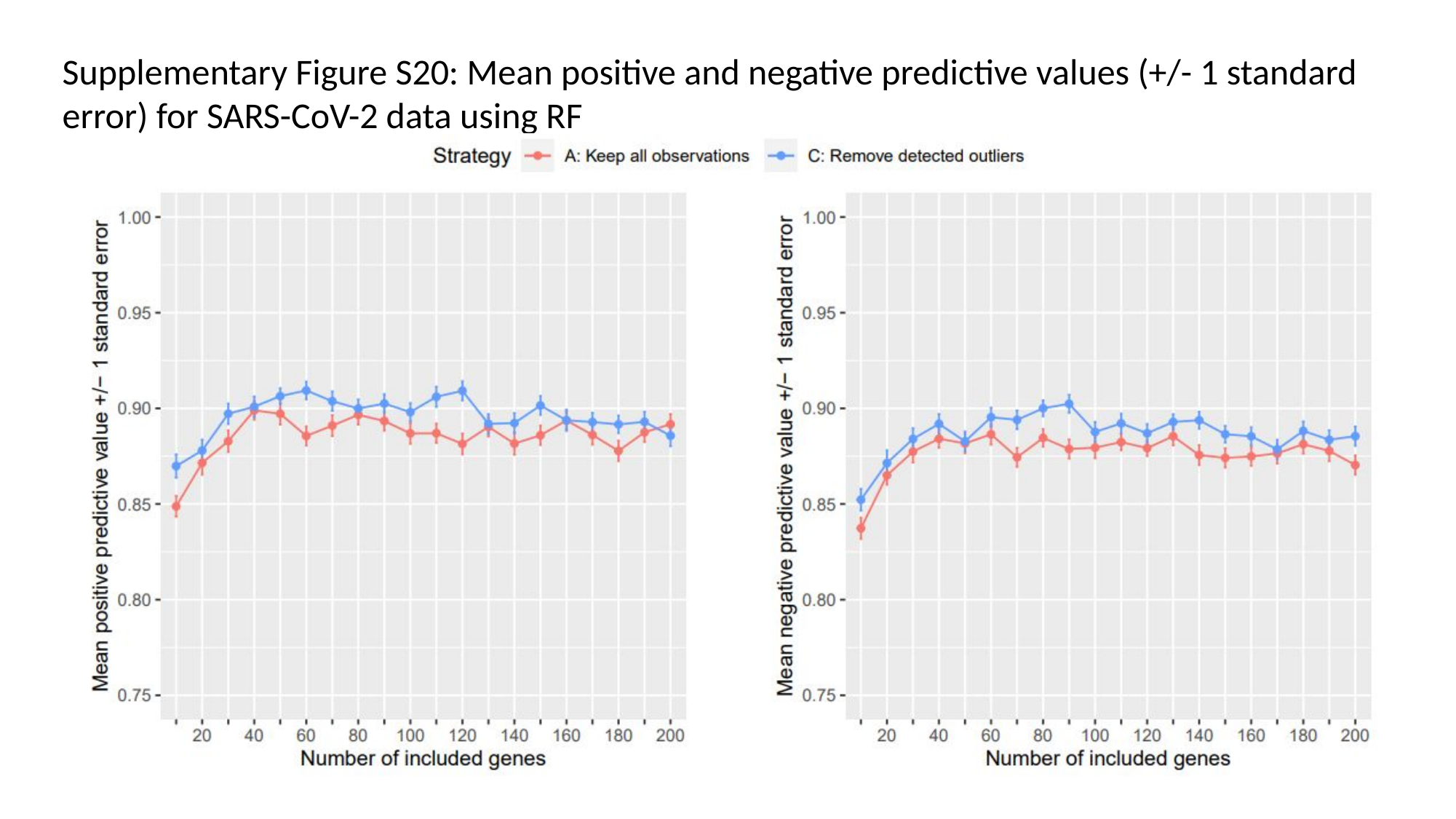

Supplementary Figure S20: Mean positive and negative predictive values (+/- 1 standard error) for SARS-CoV-2 data using RF

## Slide 22
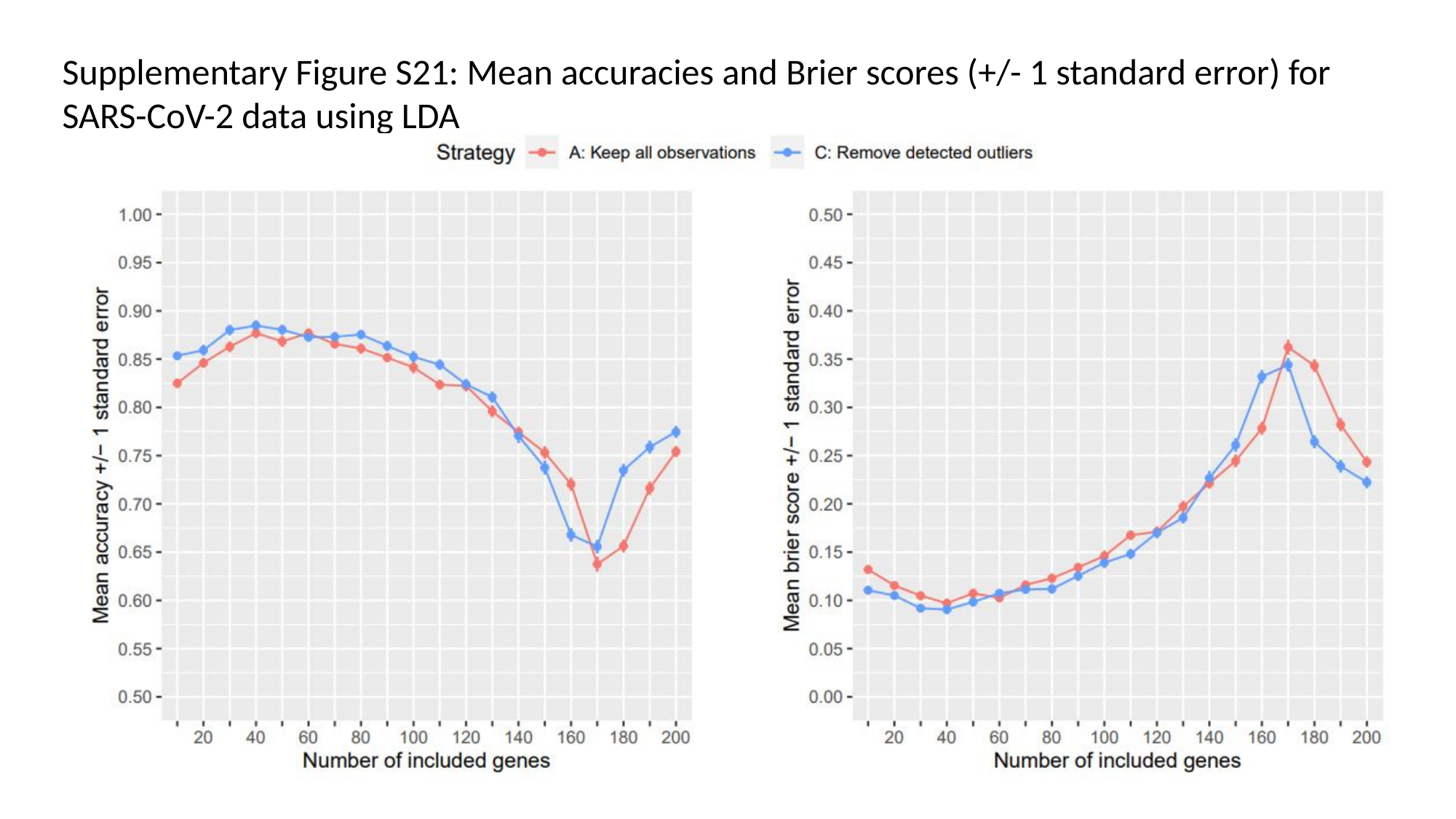

Supplementary Figure S21: Mean accuracies and Brier scores (+/- 1 standard error) for SARS-CoV-2 data using LDA

## Slide 23
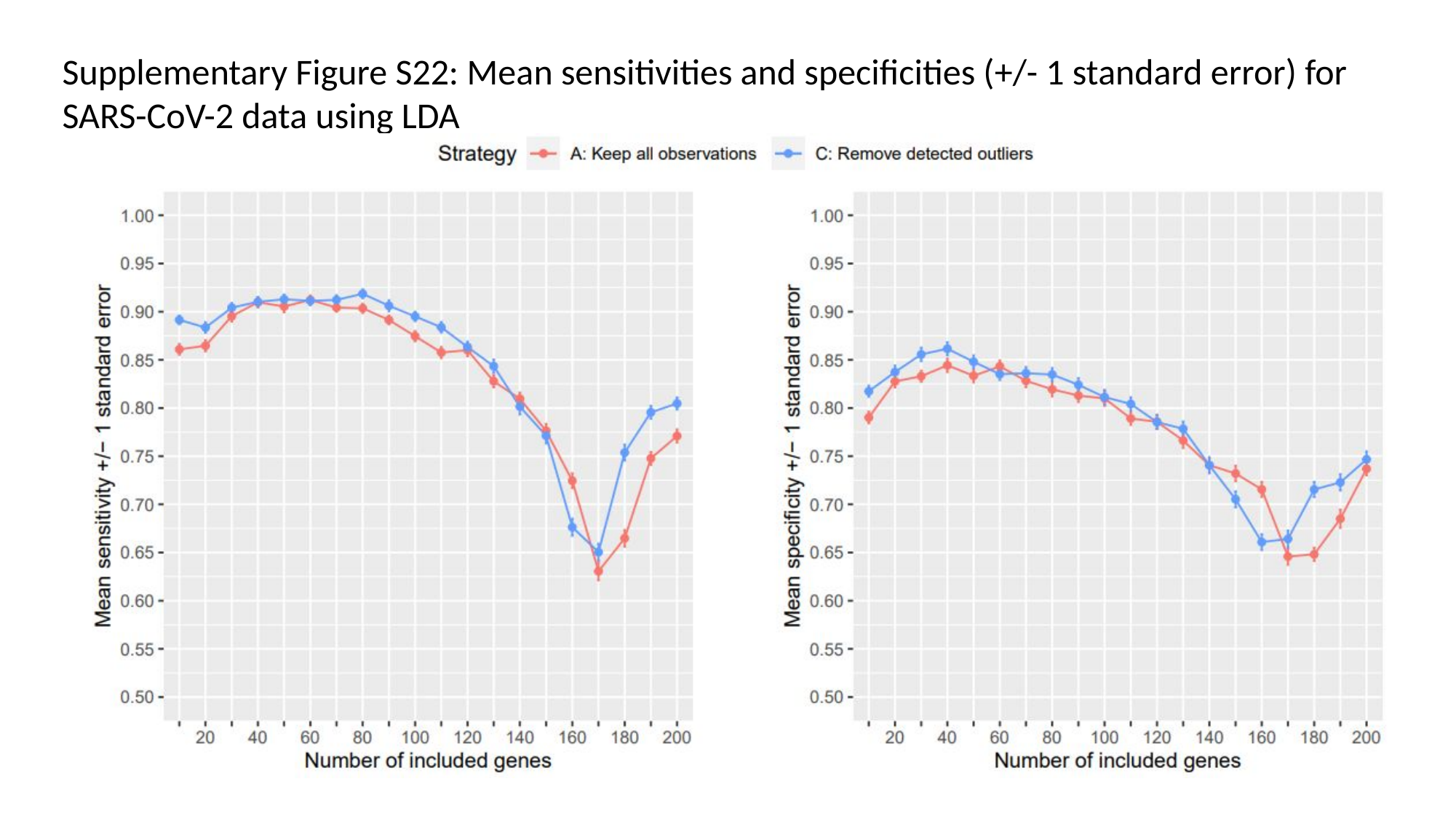

Supplementary Figure S22: Mean sensitivities and specificities (+/- 1 standard error) for SARS-CoV-2 data using LDA

## Slide 24
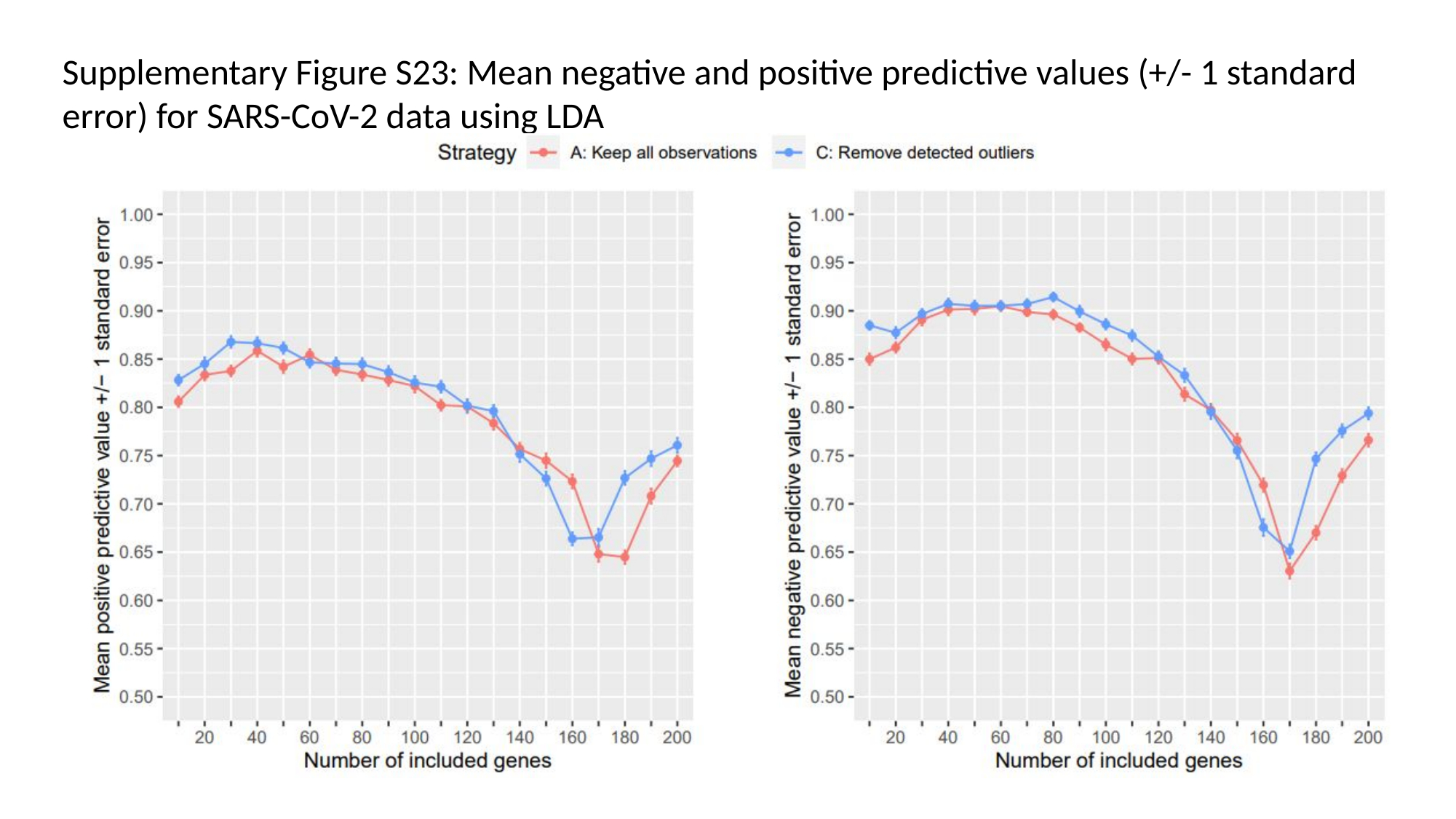

Supplementary Figure S23: Mean negative and positive predictive values (+/- 1 standard error) for SARS-CoV-2 data using LDA

## Slide 25
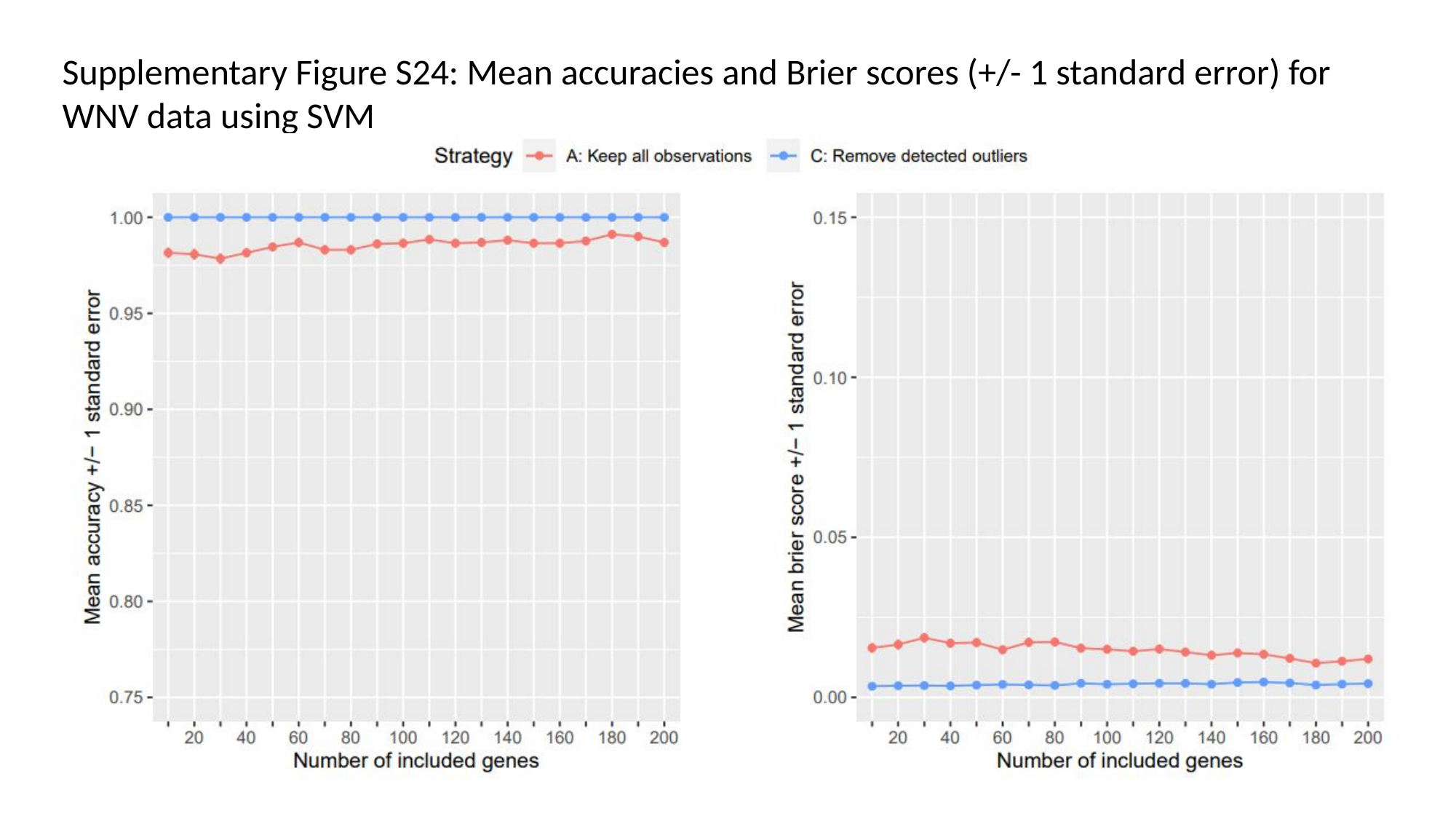

Supplementary Figure S24: Mean accuracies and Brier scores (+/- 1 standard error) for WNV data using SVM

## Slide 26
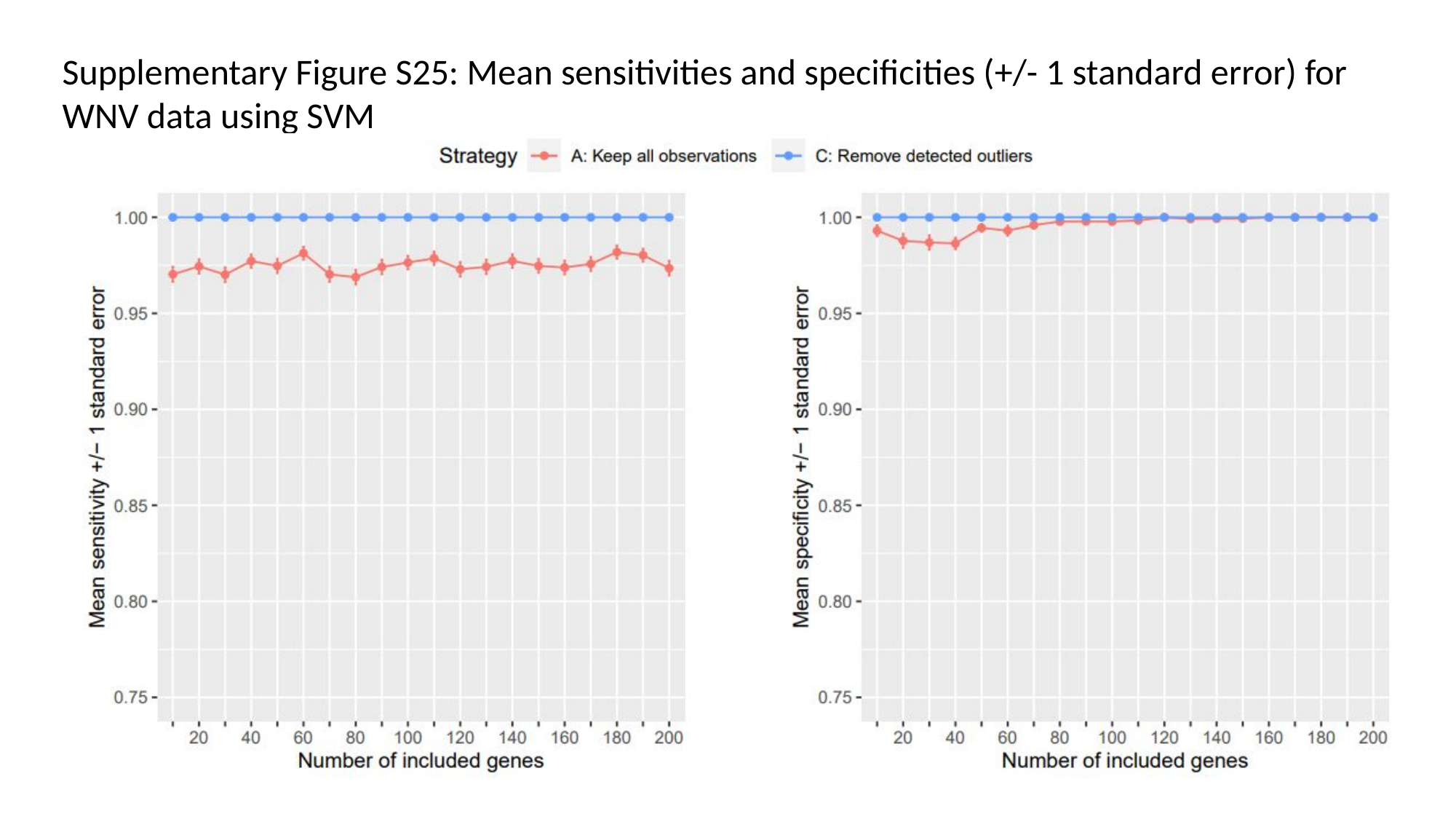

Supplementary Figure S25: Mean sensitivities and specificities (+/- 1 standard error) for WNV data using SVM

## Slide 27
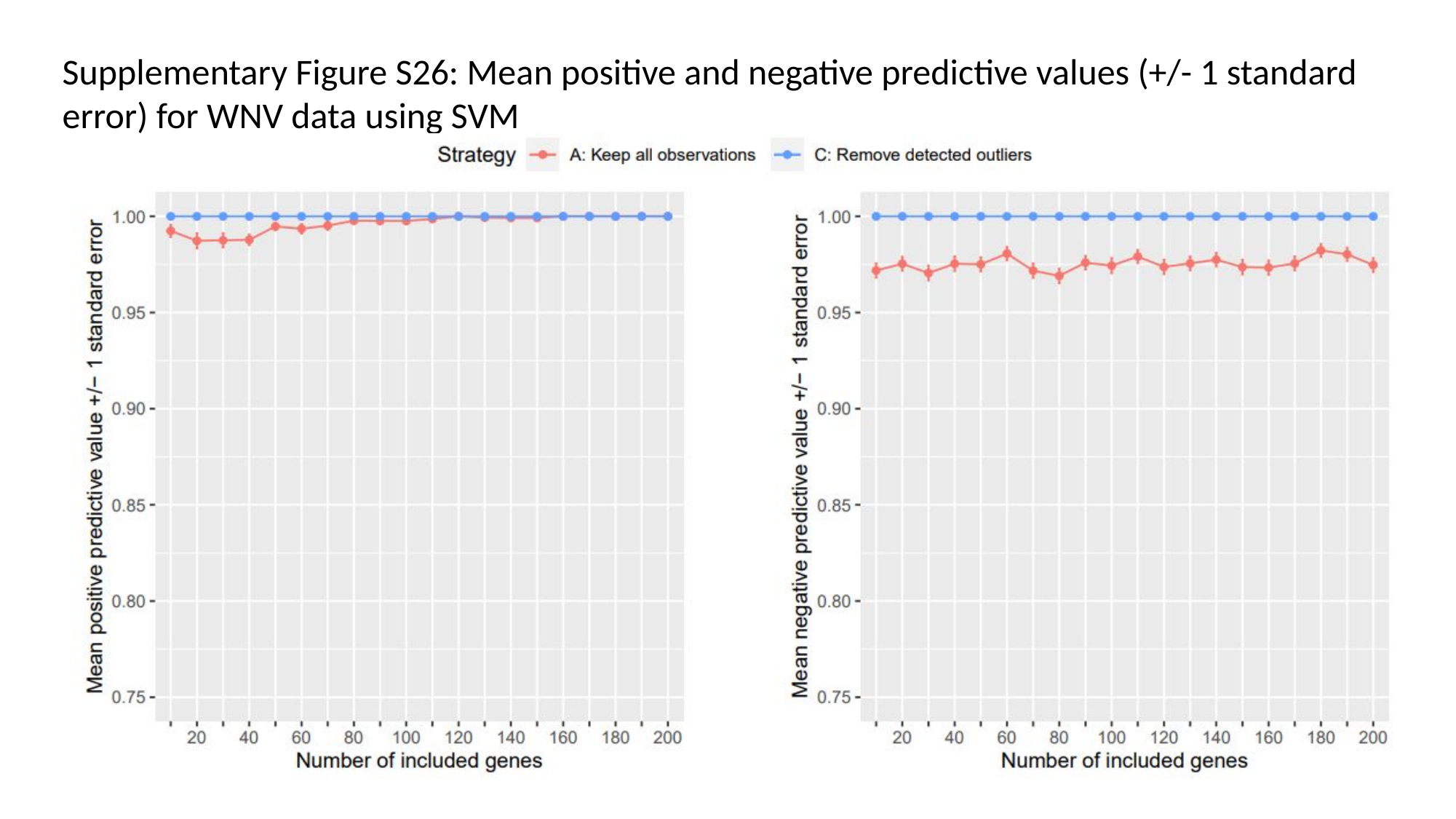

Supplementary Figure S26: Mean positive and negative predictive values (+/- 1 standard error) for WNV data using SVM

## Slide 28
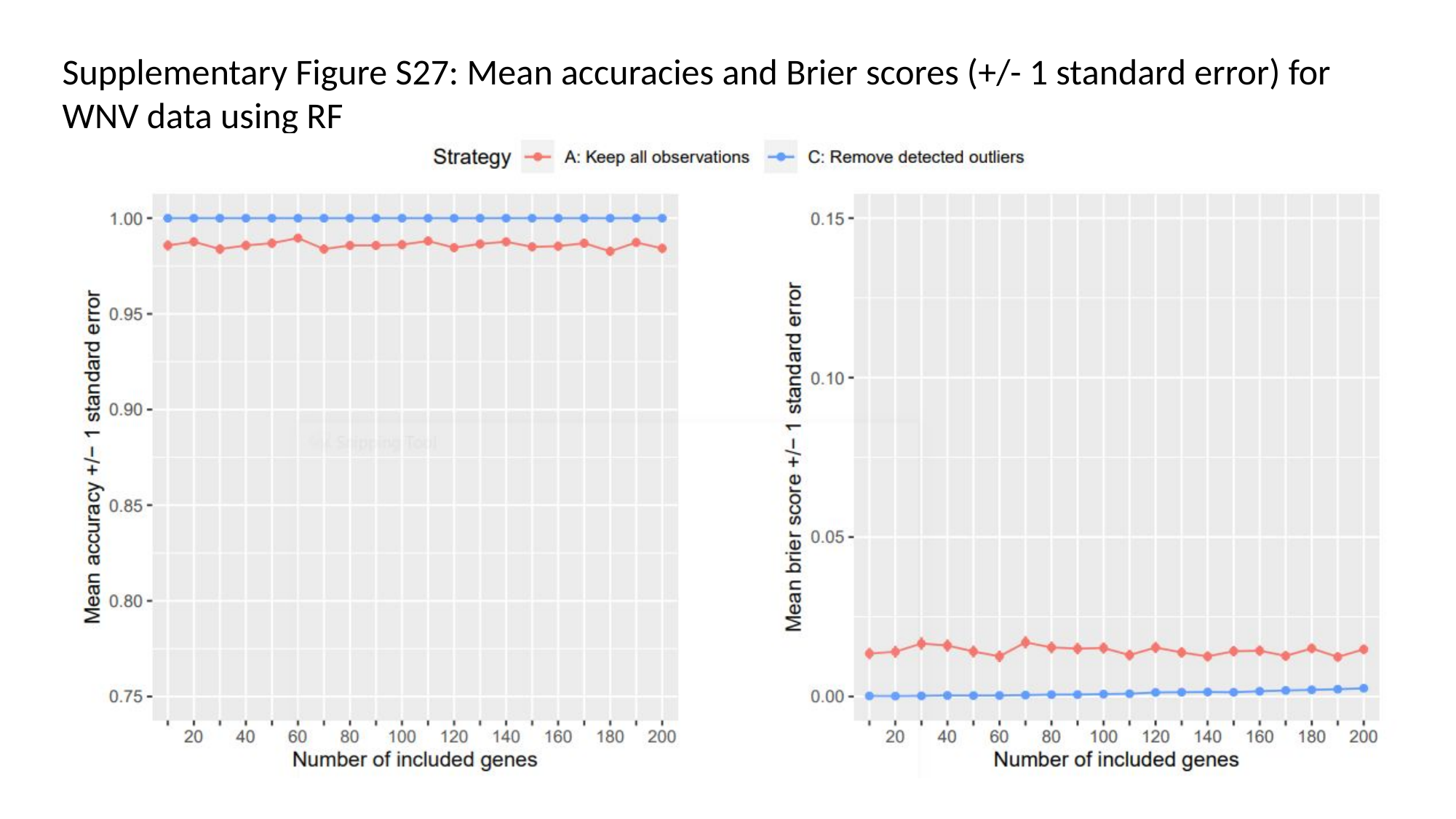

Supplementary Figure S27: Mean accuracies and Brier scores (+/- 1 standard error) for WNV data using RF

## Slide 29
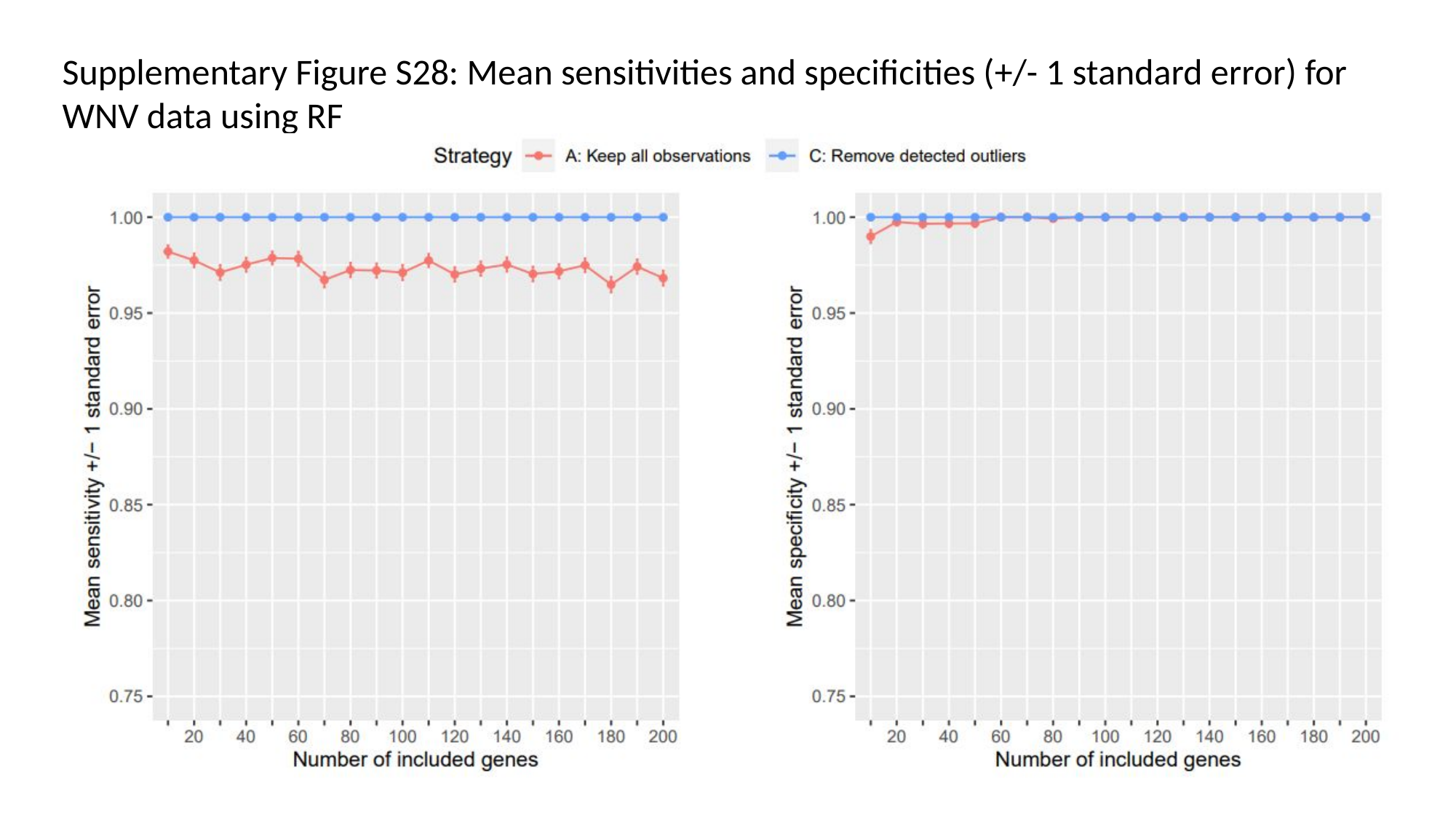

Supplementary Figure S28: Mean sensitivities and specificities (+/- 1 standard error) for WNV data using RF

## Slide 30
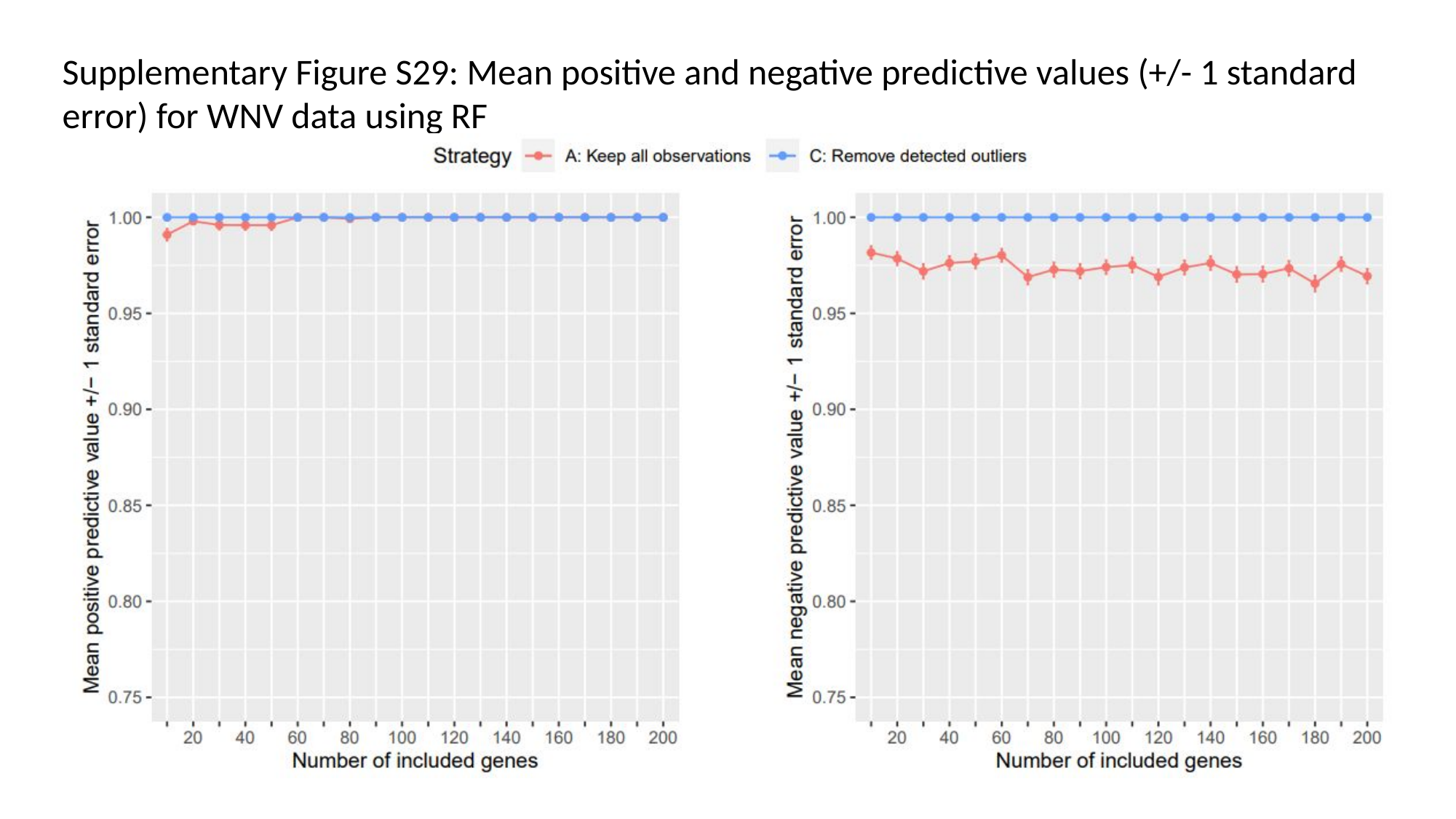

Supplementary Figure S29: Mean positive and negative predictive values (+/- 1 standard error) for WNV data using RF

## Slide 31
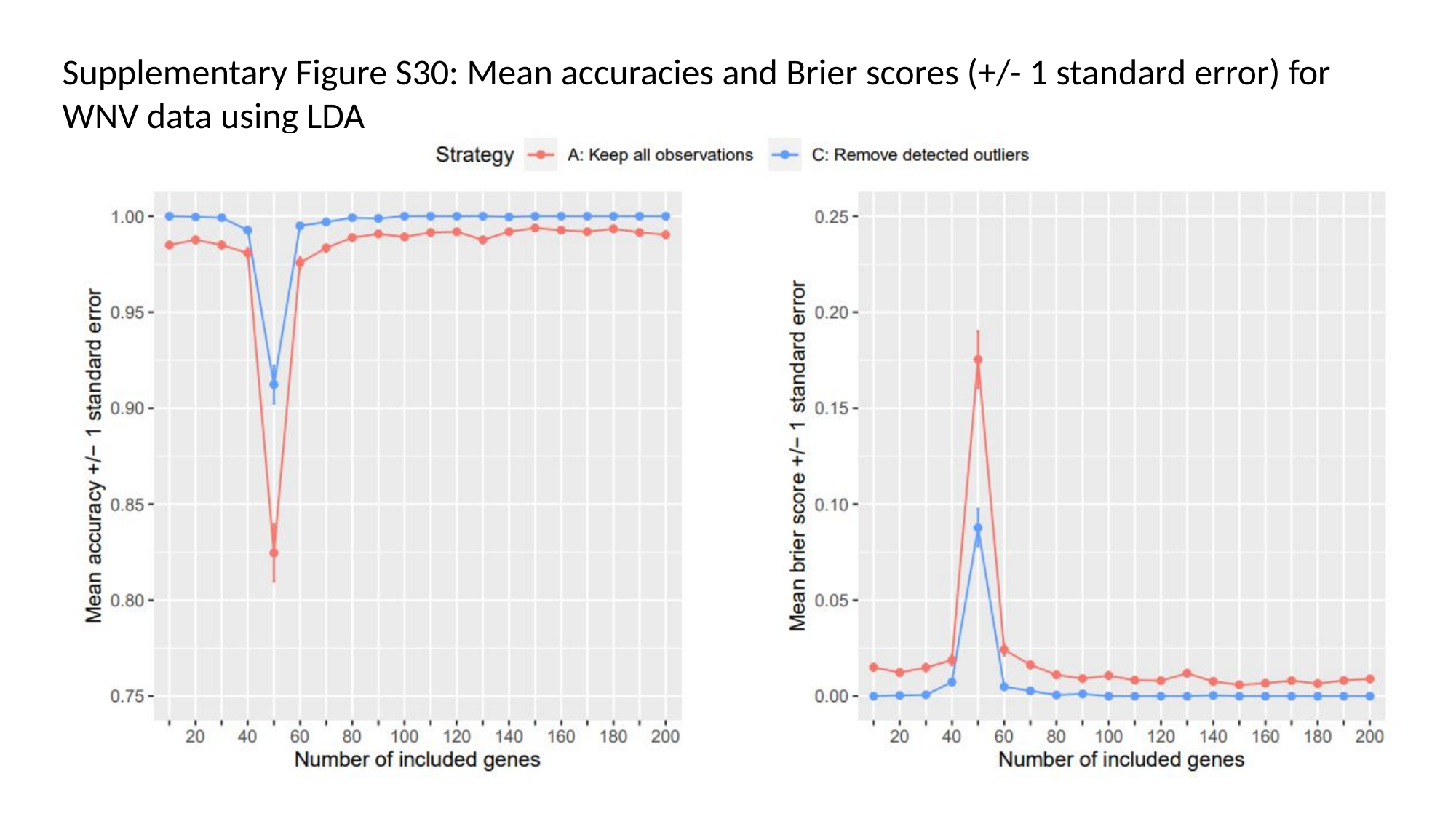

Supplementary Figure S30: Mean accuracies and Brier scores (+/- 1 standard error) for WNV data using LDA

## Slide 32
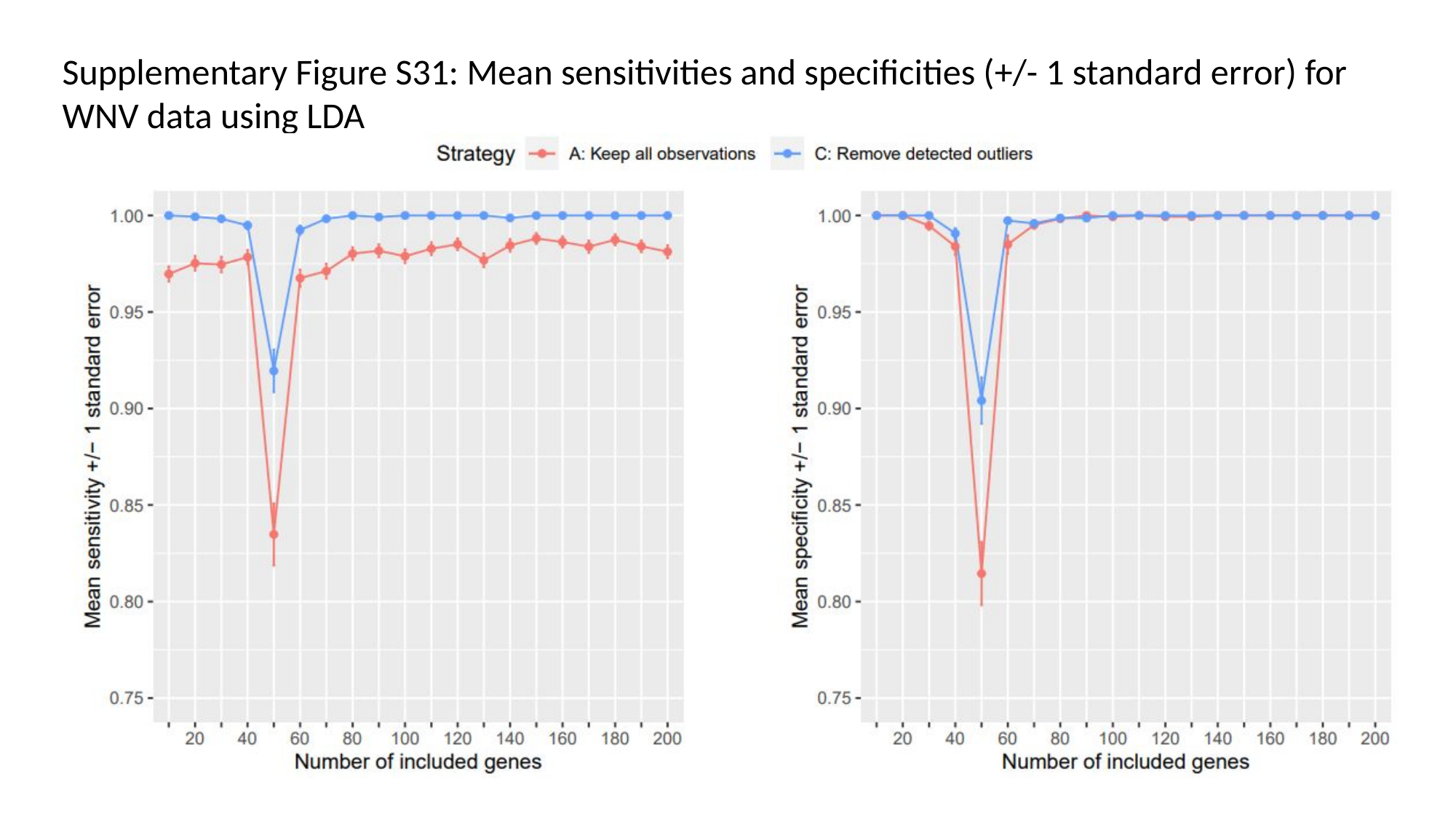

Supplementary Figure S31: Mean sensitivities and specificities (+/- 1 standard error) for WNV data using LDA

## Slide 33
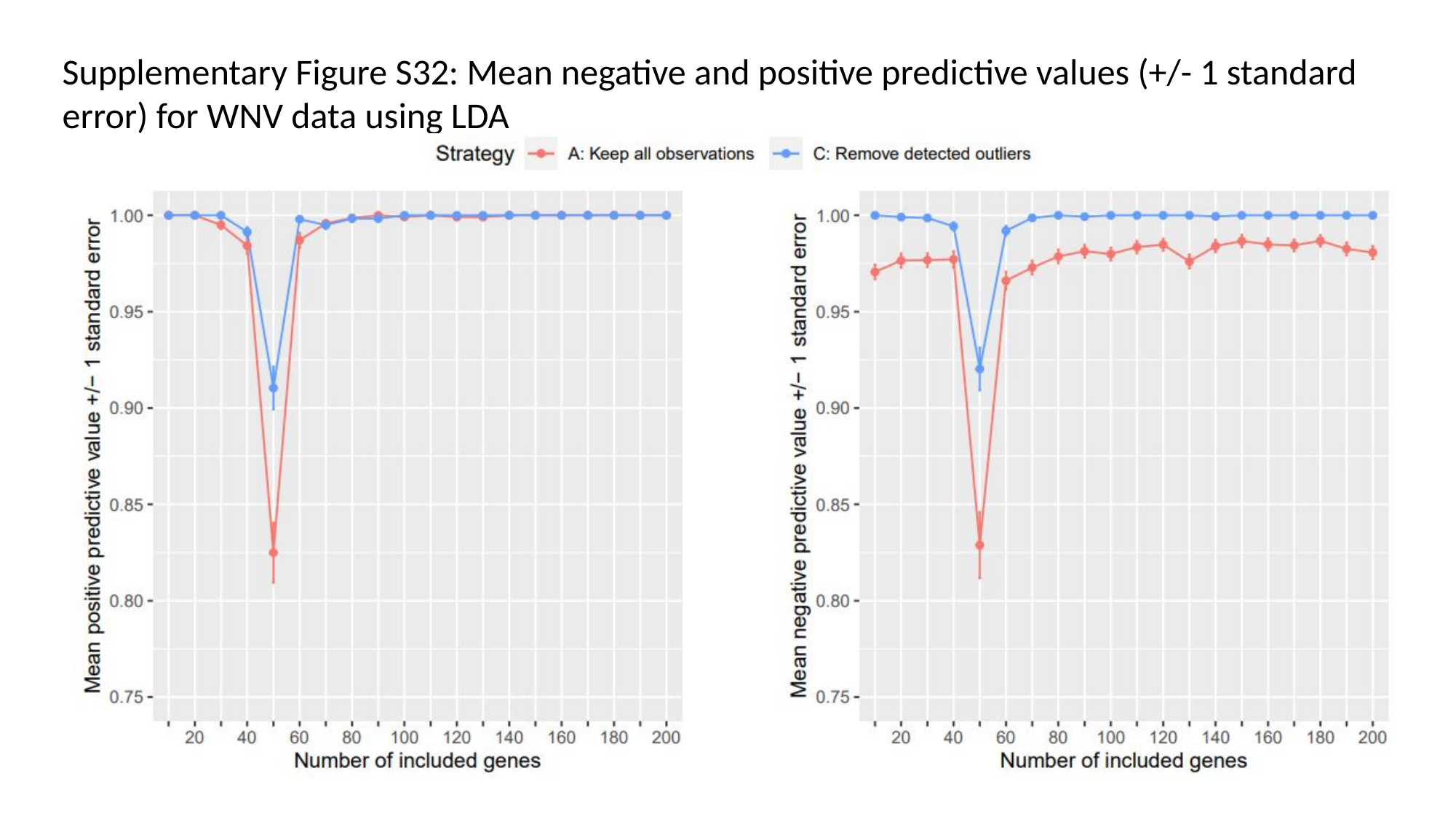

Supplementary Figure S32: Mean negative and positive predictive values (+/- 1 standard error) for WNV data using LDA

## Slide 34
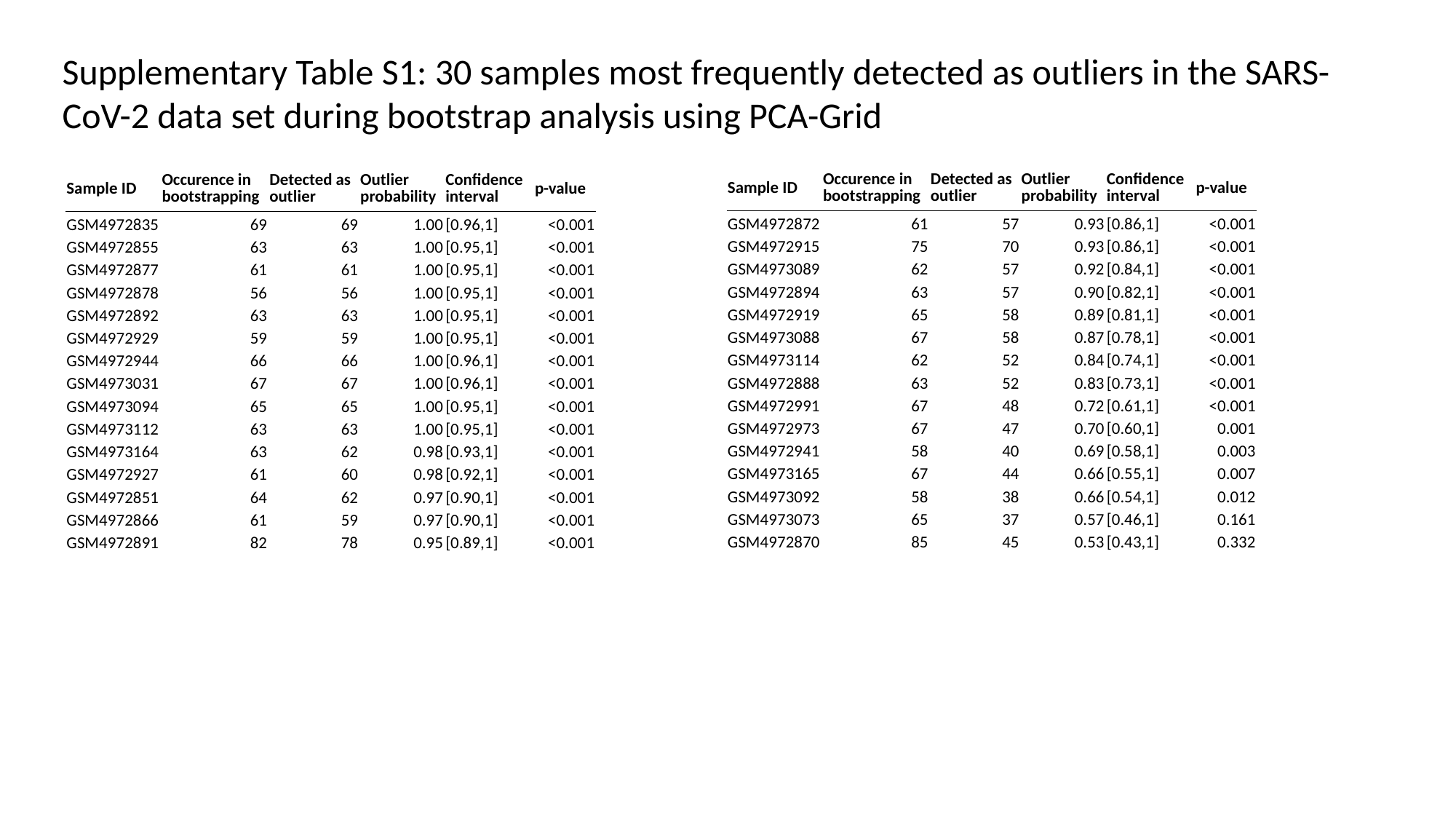

Supplementary Table S1: 30 samples most frequently detected as outliers in the SARS-CoV-2 data set during bootstrap analysis using PCA-Grid
| Sample ID | Occurence in bootstrapping | Detected as outlier | Outlier probability | Confidence interval | p-value |
| --- | --- | --- | --- | --- | --- |
| GSM4972872 | 61 | 57 | 0.93 | [0.86,1] | <0.001 |
| GSM4972915 | 75 | 70 | 0.93 | [0.86,1] | <0.001 |
| GSM4973089 | 62 | 57 | 0.92 | [0.84,1] | <0.001 |
| GSM4972894 | 63 | 57 | 0.90 | [0.82,1] | <0.001 |
| GSM4972919 | 65 | 58 | 0.89 | [0.81,1] | <0.001 |
| GSM4973088 | 67 | 58 | 0.87 | [0.78,1] | <0.001 |
| GSM4973114 | 62 | 52 | 0.84 | [0.74,1] | <0.001 |
| GSM4972888 | 63 | 52 | 0.83 | [0.73,1] | <0.001 |
| GSM4972991 | 67 | 48 | 0.72 | [0.61,1] | <0.001 |
| GSM4972973 | 67 | 47 | 0.70 | [0.60,1] | 0.001 |
| GSM4972941 | 58 | 40 | 0.69 | [0.58,1] | 0.003 |
| GSM4973165 | 67 | 44 | 0.66 | [0.55,1] | 0.007 |
| GSM4973092 | 58 | 38 | 0.66 | [0.54,1] | 0.012 |
| GSM4973073 | 65 | 37 | 0.57 | [0.46,1] | 0.161 |
| GSM4972870 | 85 | 45 | 0.53 | [0.43,1] | 0.332 |
| Sample ID | Occurence in bootstrapping | Detected as outlier | Outlier probability | Confidence interval | p-value |
| --- | --- | --- | --- | --- | --- |
| GSM4972835 | 69 | 69 | 1.00 | [0.96,1] | <0.001 |
| GSM4972855 | 63 | 63 | 1.00 | [0.95,1] | <0.001 |
| GSM4972877 | 61 | 61 | 1.00 | [0.95,1] | <0.001 |
| GSM4972878 | 56 | 56 | 1.00 | [0.95,1] | <0.001 |
| GSM4972892 | 63 | 63 | 1.00 | [0.95,1] | <0.001 |
| GSM4972929 | 59 | 59 | 1.00 | [0.95,1] | <0.001 |
| GSM4972944 | 66 | 66 | 1.00 | [0.96,1] | <0.001 |
| GSM4973031 | 67 | 67 | 1.00 | [0.96,1] | <0.001 |
| GSM4973094 | 65 | 65 | 1.00 | [0.95,1] | <0.001 |
| GSM4973112 | 63 | 63 | 1.00 | [0.95,1] | <0.001 |
| GSM4973164 | 63 | 62 | 0.98 | [0.93,1] | <0.001 |
| GSM4972927 | 61 | 60 | 0.98 | [0.92,1] | <0.001 |
| GSM4972851 | 64 | 62 | 0.97 | [0.90,1] | <0.001 |
| GSM4972866 | 61 | 59 | 0.97 | [0.90,1] | <0.001 |
| GSM4972891 | 82 | 78 | 0.95 | [0.89,1] | <0.001 |

## Slide 35
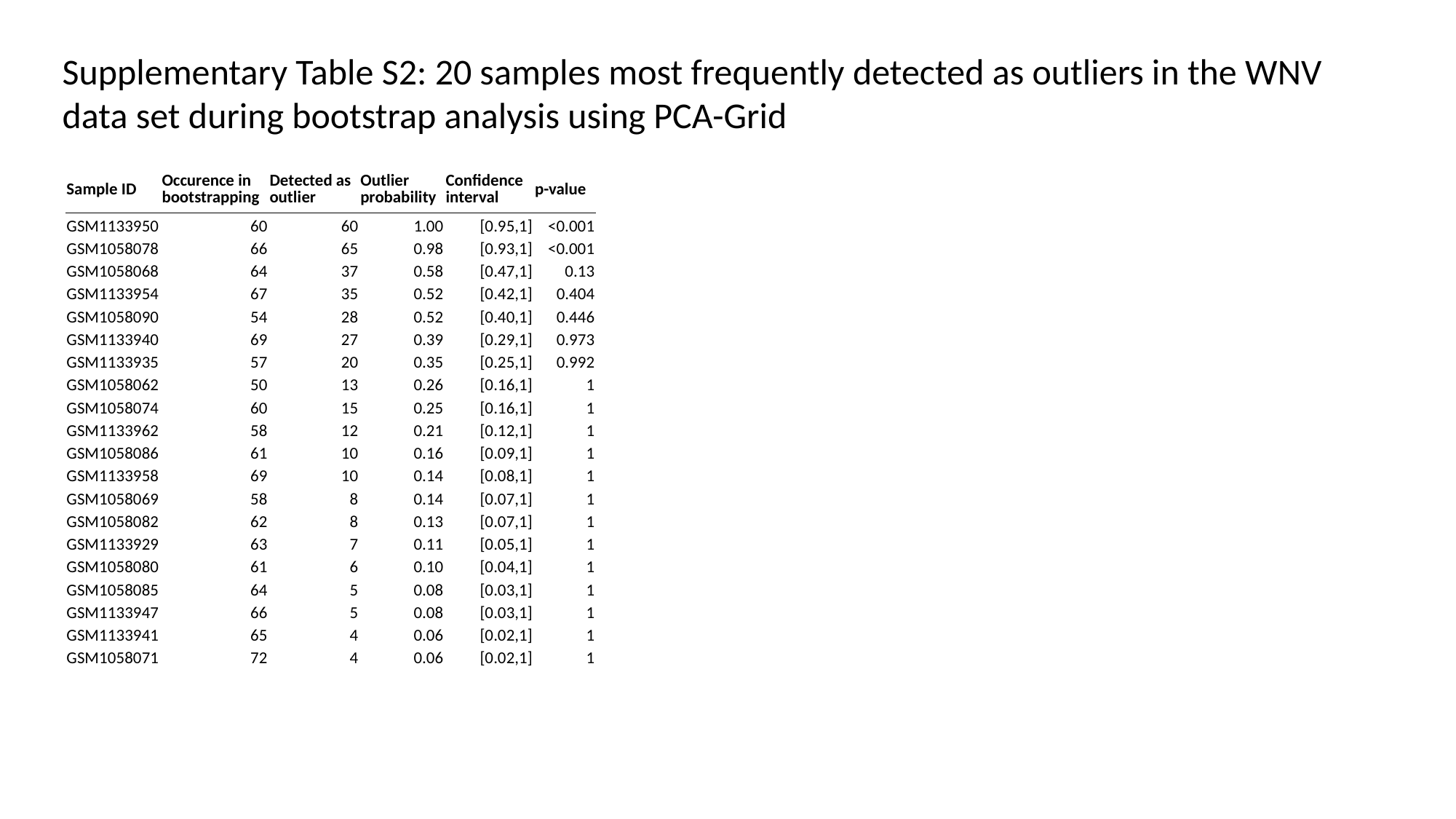

Supplementary Table S2: 20 samples most frequently detected as outliers in the WNV data set during bootstrap analysis using PCA-Grid
| Sample ID | Occurence in bootstrapping | Detected as outlier | Outlier probability | Confidence interval | p-value |
| --- | --- | --- | --- | --- | --- |
| GSM1133950 | 60 | 60 | 1.00 | [0.95,1] | <0.001 |
| GSM1058078 | 66 | 65 | 0.98 | [0.93,1] | <0.001 |
| GSM1058068 | 64 | 37 | 0.58 | [0.47,1] | 0.13 |
| GSM1133954 | 67 | 35 | 0.52 | [0.42,1] | 0.404 |
| GSM1058090 | 54 | 28 | 0.52 | [0.40,1] | 0.446 |
| GSM1133940 | 69 | 27 | 0.39 | [0.29,1] | 0.973 |
| GSM1133935 | 57 | 20 | 0.35 | [0.25,1] | 0.992 |
| GSM1058062 | 50 | 13 | 0.26 | [0.16,1] | 1 |
| GSM1058074 | 60 | 15 | 0.25 | [0.16,1] | 1 |
| GSM1133962 | 58 | 12 | 0.21 | [0.12,1] | 1 |
| GSM1058086 | 61 | 10 | 0.16 | [0.09,1] | 1 |
| GSM1133958 | 69 | 10 | 0.14 | [0.08,1] | 1 |
| GSM1058069 | 58 | 8 | 0.14 | [0.07,1] | 1 |
| GSM1058082 | 62 | 8 | 0.13 | [0.07,1] | 1 |
| GSM1133929 | 63 | 7 | 0.11 | [0.05,1] | 1 |
| GSM1058080 | 61 | 6 | 0.10 | [0.04,1] | 1 |
| GSM1058085 | 64 | 5 | 0.08 | [0.03,1] | 1 |
| GSM1133947 | 66 | 5 | 0.08 | [0.03,1] | 1 |
| GSM1133941 | 65 | 4 | 0.06 | [0.02,1] | 1 |
| GSM1058071 | 72 | 4 | 0.06 | [0.02,1] | 1 |
